# Therapeutic Potential of Marine Natural Products Against Alzheimer’s Disease: Molecular Mechanisms, Druggability Evaluation and Clinical Translation Barriers

**DOI:** 10.3390/md24100356

**Published:** 2026-10-10

**Authors:** Daowei Huang, Roujun Lu, Chenchen Sheng, Jie Zheng, Yafei Wang, Zhipei Sang

**Affiliations:** 1 School of Chemical and Pharmaceutical Engineering, Hebei University of Science and Technology, Shijiazhuang 050018, China; 13373855814@163.com (C.S.); zj13012002851@163.com (J.Z.); 15231023115@163.com (Y.W.); 2 Key Laboratory of Tropical Biological Resources of Ministry of Education and Hainan Engineering Research Center for Drug Screening and Evaluation, School of Pharmaceutical Sciences, Hainan University, Haikou 570228, China; 25221055000026@hainanu.edu.cn

**Keywords:** marine natural products, Alzheimer’s disease, multi-target neuroprotective, ADMET, blood–brain barrier, gut-brain axis, clinical translation

## Abstract

Alzheimer’s disease (AD) is an irreversible neurodegenerative disorder with complex multifactorial pathological cascades, including amyloid-β (Aβ) aggregation, tau hyperphosphorylation, sustained neuroinflammation, oxidative stress, cholinergic dysfunction and gut–brain axis imbalance. Current marketed anti-AD drugs merely relieve symptomatic cognitive impairment without halting or reversing disease progression, highlighting an urgent demand for novel multi-target therapeutic candidates. Marine ecosystems, covering 71% of the Earth’s surface, generate structurally unique, low-toxicity secondary metabolites via adaptive metabolic pathways of marine algae, invertebrates and microorganisms, which represent promising lead compounds for developing marine anti-AD therapeutics with synergistic multitarget neuroprotective activities. This systematic review comprehensively summarizes the anti-AD bioactivities and underlying molecular mechanisms of seven major categories of marine natural products, including marine polysaccharides, terpenoids, macrolides, alkaloids, polyphenols and carotenoids, sterols/lipids, and aminosulfonates. We further perform an integrated ADMET druggability assessment of representative marine bioactive molecules to evaluate their blood–brain barrier penetration, oral absorption, physicochemical properties and toxicological risks, identifying **homotaurine** as the most balanced candidate with favorable central nervous system delivery and safety profiles. We also elaborate the core bottlenecks restricting clinical translation, including insufficient blood–brain barrier permeability and unsatisfactory in vivo pharmacokinetic behaviors of most marine-derived compounds. Finally, we outline future research directions, covering structural modification, nanocarrier delivery, gut–brain axis-targeted intervention and cross-disciplinary translational research. This work aims to provide a comprehensive theoretical reference for the discovery, optimization and clinical transformation of marine anti-Alzheimer drugs, and facilitate high-value exploitation of marine bioactive resources for neurodegenerative disease treatment.

## 1. Introduction

With the accelerating global population aging, the incidence of Alzheimer’s disease (AD) rises year by year, evolving into a critical public health issue threatening the health and quality of life of the elderly. Statistics show that there are more than 50 million AD patients worldwide, and this figure is projected to exceed 150 million by 2050 [1,2], imposing a heavy burden on families, society and medical systems. As an irreversible neurodegenerative disorder, AD represents the predominant form of dementia [3]. It is characterized by progressive degeneration of neurons, particularly those in the cholinergic system, which manifests as memory impairment, confusion and cognitive decline [4,5], posing a severe health threat to the elderly. More than a century has passed since AD was first reported by Alzheimer in 1906. Despite massive scientific research investment, effective therapies capable of slowing or halting disease progression remain unavailable [6]. The pathological mechanism of AD is extremely complex and not fully elucidated. It is generally acknowledged that AD arises from the combined effects of genetic, environmental, metabolic and other factors. Its core pathological hallmarks consist of abnormal aggregation of amyloid-β (Aβ) peptides forming senile plaques, hyperphosphorylation of tau protein leading to neurofibrillary tangles, damage to cholinergic neurons [7], persistent neuroinflammation and oxidative stress imbalance [8].

At the molecular level, the pathogenic cascade of AD is intimately linked to protein misfolding and aberrant aggregation. Under pathological conditions, amyloid-β monomers undergo conformational transitions from random coil to β-sheet-rich structures, a process that initiates the formation of neurotoxic oligomers and subsequently mature fibrils [9,10]. These findings highlight the importance of understanding the nucleation and aggregation pathways of amyloidogenic proteins, and suggest that interfering with these early conformational transitions represents a rational therapeutic strategy for AD intervention.

**Figure 1:**
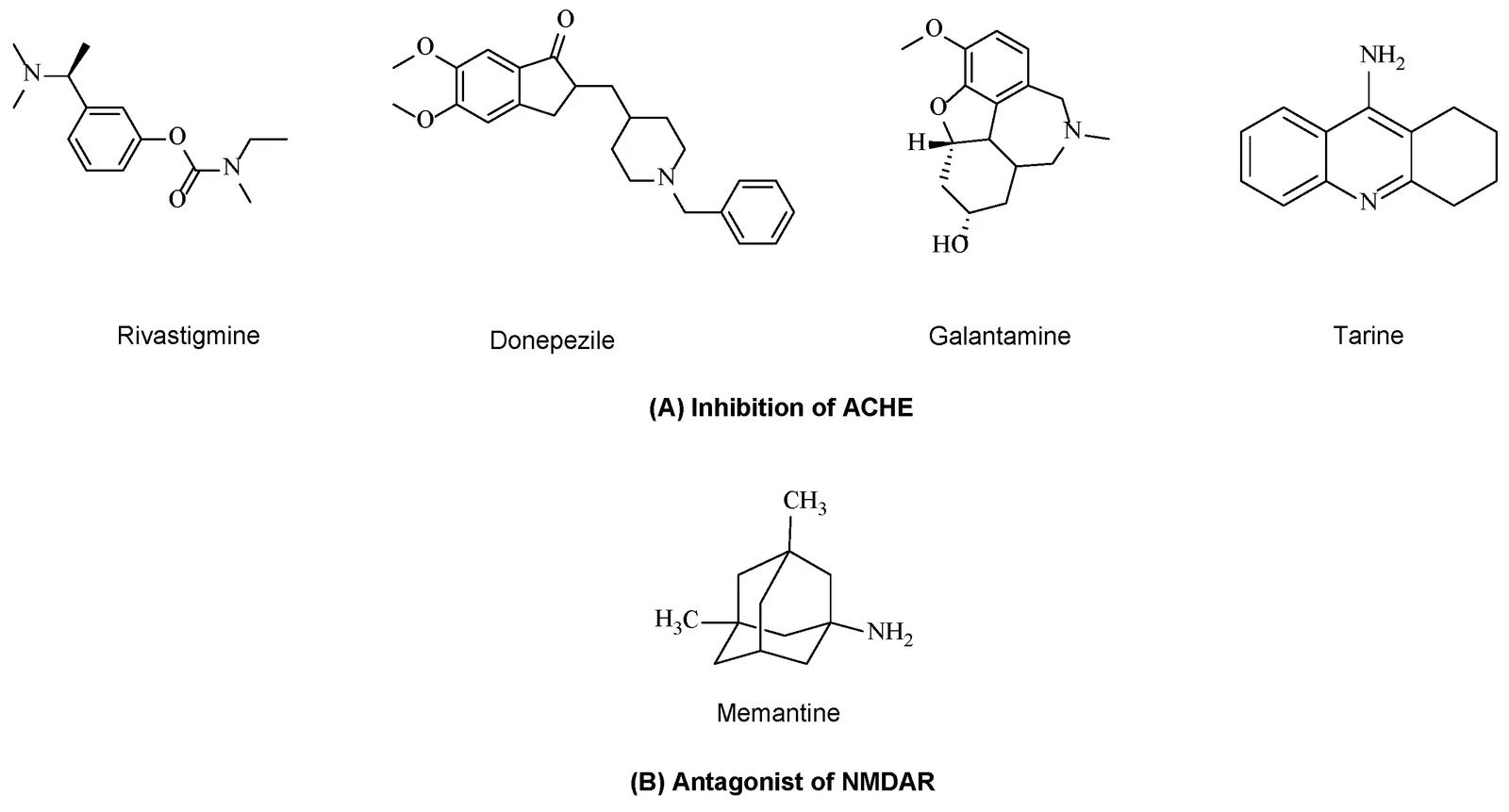
Representative drugs for Alzheimer’s disease. (**A**) Acetylcholinesterase (AChE) inhibitors, (**B**) N-methyl-D-aspartate receptor (NMDAR) antagonist.

**Figure 2:**
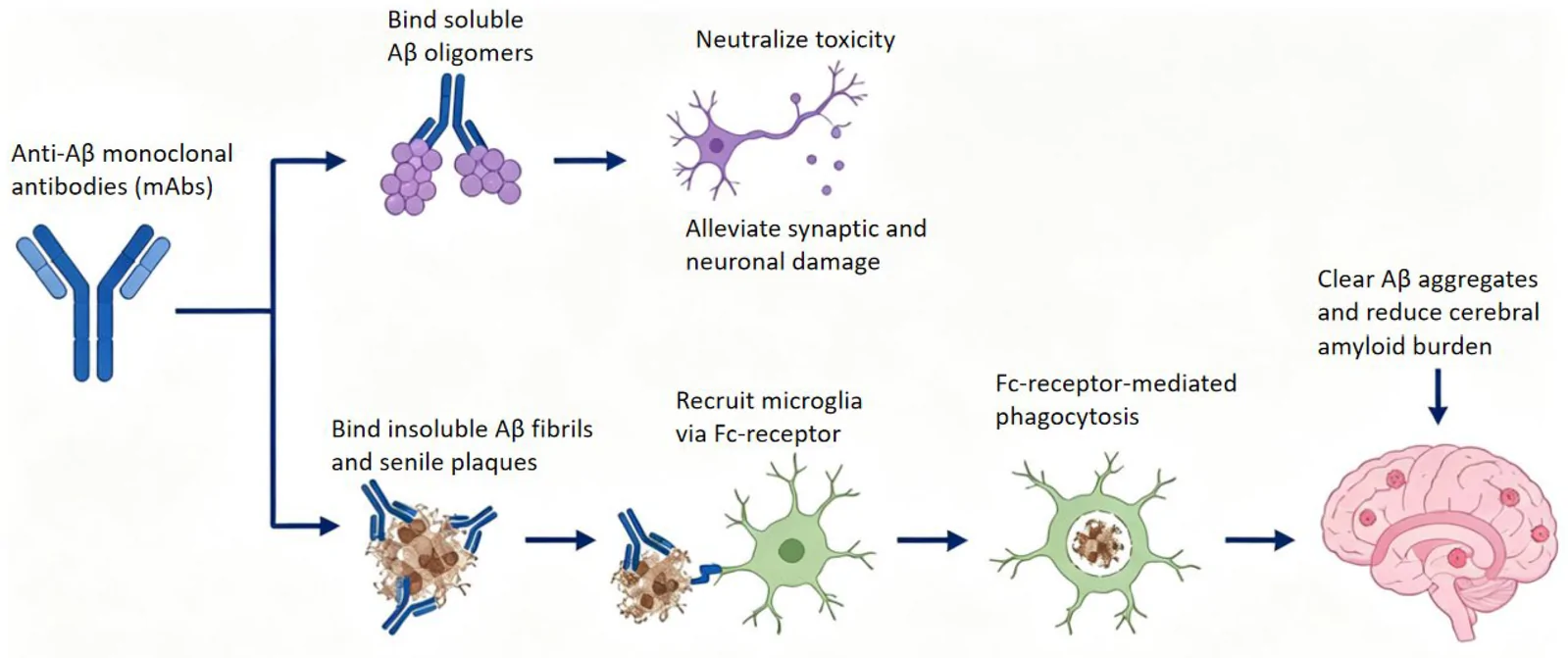
Mechanism of action of monoclonal antibodies against Alzheimer’s disease (AD).

Despite substantial research efforts devoted to Alzheimer’s disease, the definitive etiology remains incompletely elucidated, and available therapeutic strategies exhibit limited potency in retarding disease progression and ameliorating clinical symptoms. Cholinesterase inhibitors constitute the predominant class of clinically approved anti-AD agents. Such drugs suppress acetylcholinesterase (AChE) activity to inhibit the degradation of acetylcholine (ACh), elevate cerebral ACh concentration, and thereby mitigate cognitive impairment [11]. Typical representatives include rivastigmine, donepezil, galantamine and tacrine (small molecules, Figure 1, Table 2). These compounds can alleviate clinical manifestations in some patients, whereas they fail to fundamentally intervene the core pathological cascade with modest overall therapeutic outcomes. Memantine (small-molecule, Table 2), an N-methyl-D-aspartate (NMDA) receptor antagonist [12], alleviates excitotoxic neuronal injury and preserves neuronal viability via restraining excessive activation of NMDA receptors, as illustrated in Figure 1. In addition to the aforementioned small-molecule symptomatic agents, monoclonal antibodies targeting amyloid-β (Aβ) have emerged as a breakthrough class of disease-modifying therapies for Alzheimer’s disease (AD). Aducanumab (Table 2), as the first FDA-approved antibody of this class, facilitates the clearance of cerebral Aβ plaques via microglia-mediated phagocytosis; however, its clinical benefit remains controversial. Lecanemab, (Table 2) which received full FDA approval in 2023, preferentially binds to soluble Aβ protofibrils and demonstrated a 27% reduction in clinical decline as measured by the CDR-SB scale in its Phase III Clarity AD trial [13,14]. Donanemab (Table 2), which targets pyroglutamate-modified Aβ, has also shown significant deceleration of cognitive and functional decline in early-stage AD patients [15]. Collectively, these antibodies provide compelling validation of the amyloid cascade hypothesis and represent therapeutics that mechanistically intervene in the core pathological processes of AD (Figure 2). However, their clinical application is constrained by notable limitations, including high costs, intravenous administration, and restriction to early-stage patients, whereas their efficacy in moderate-to-severe AD populations and long-term safety profiles remain to be fully established. Nevertheless, these medications merely alleviate clinical symptoms of AD patients, failing to target core pathological pathways. They are incapable of arresting or reversing disease progression. Moreover, long-term administration may trigger adverse reactions including gastrointestinal disorders, dizziness and insomnia. Accordingly, the development of novel anti-AD therapeutics remains a formidable challenge, and the identification of innovative therapeutic targets and intervention strategies has emerged as an urgent research priority.

In recent years, driven by the rapid advancement of separation and purification techniques, medicinal chemistry and molecular biology, an increasing number of marine natural products have been confirmed to possess definite anti-AD bioactivity. Several candidate compounds have advanced into preclinical and clinical research stages, offering novel insights and therapeutic strategies for AD treatment. This review systematically summarizes the research progress of diverse marine natural products against Alzheimer’s disease, analyzes their underlying action mechanisms and existing limitations, and prospects future research trends, aiming to provide theoretical foundations for subsequent relevant investigations.

Marine natural products play an indispensable role in pharmaceutical research and development. Subjected to extreme environmental conditions including high hydrostatic pressure, high salinity, low temperature and hypoxia, marine organisms such as algae, sponges, corals and marine microorganisms have evolved metabolic systems distinctly different from their terrestrial counterparts [16]. These organisms synthesize abundant secondary metabolites with novel skeletons and diverse biological activities, covering polysaccharides, terpenoids, alkaloids, polyphenols and steroids. Possessing multi-target synergistic effects, marine natural products can modulate multiple pathological processes of AD, including amyloid-β aggregation, tau hyperphosphorylation, neuroinflammation and oxidative stress. Combined with low toxicity and favorable biocompatibility [17], they hold great prospective value for the development of anti-Alzheimer agents.

## 2. Core Mechanisms of Marine Natural Products Against Alzheimer’s Disease

The pathological progression of AD arises from synergistic interactions of multiple cascades and targets, and single-target intervention hardly achieves satisfactory therapeutic efficacy. Owing to structural diversity, marine natural products can collaboratively modulate core pathological processes of AD via diverse pathways. Their predominant functional mechanisms are categorized into seven aspects, as detailed below.

### 2.1. Inhibition of Aβ Production, Aggregation and Deposition

**Figure 3:**
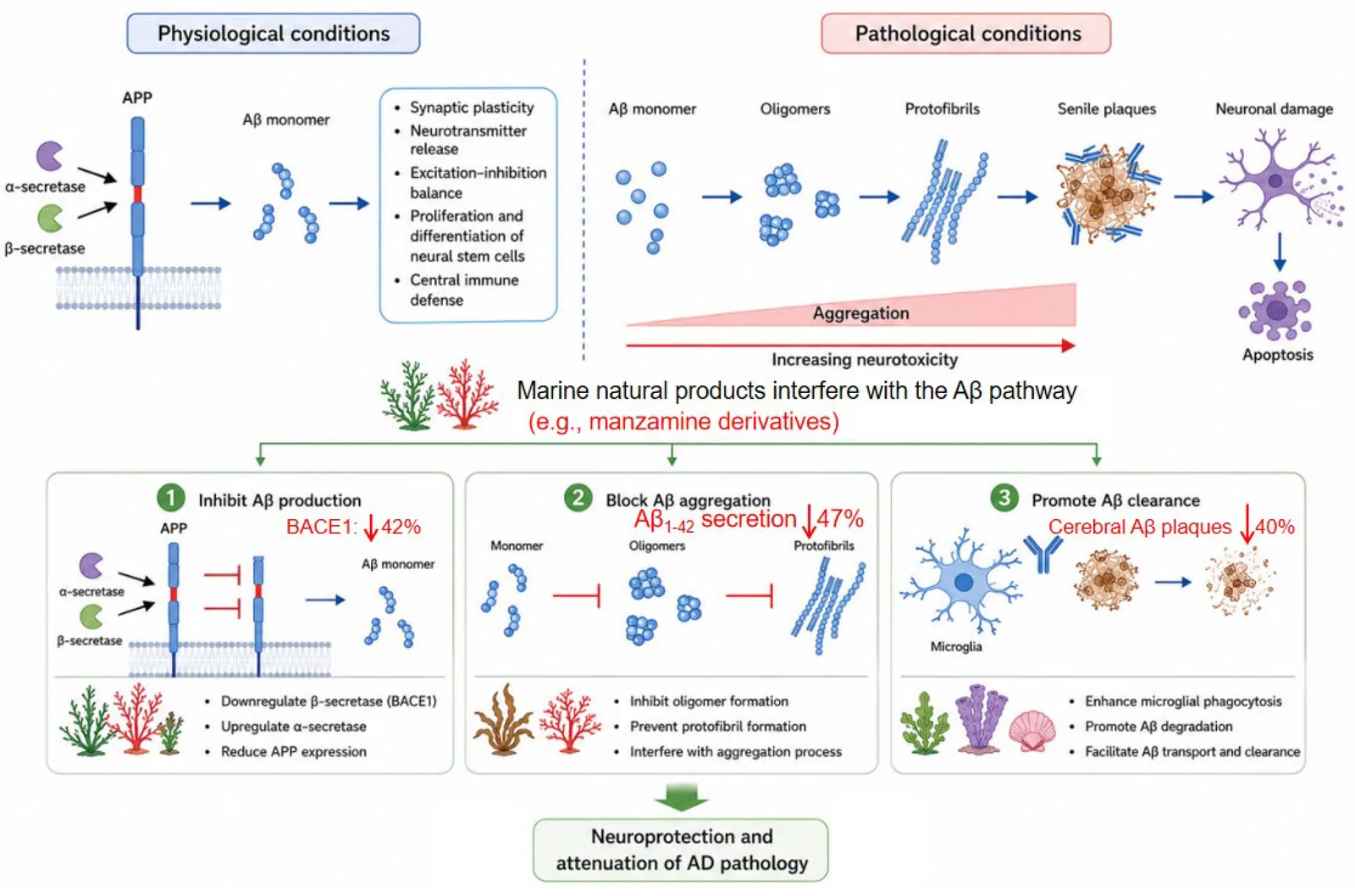
Pathological process of abnormal Aβ aggregation and intervention mechanisms of marine natural products.

Under physiological conditions, Aβ is an endogenous peptide produced via secretase-mediated proteolytic hydrolysis of amyloid-β precursor protein (APP). Its monomer participates in multiple processes including synaptic plasticity, neurotransmitter release, excitation–inhibition balance, and proliferation and differentiation of neural stem cells, as well as a central immune defense [18]. Senile plaques resulting from abnormal aggregation of Aβ represent one of the most typical pathological hallmarks of AD. Under pathological conditions, Aβ monomers gradually aggregate into highly neurotoxic oligomers and protofibrils, and eventually deposit to form senile plaques, thereby inducing neuronal damage and apoptosis [19]. Marine natural products interfere with the Aβ pathway via three approaches, namely inhibiting Aβ production, blocking its aggregation and promoting its clearance so as to exert neuroprotective effects (Figure 3).

**Figure 10:**
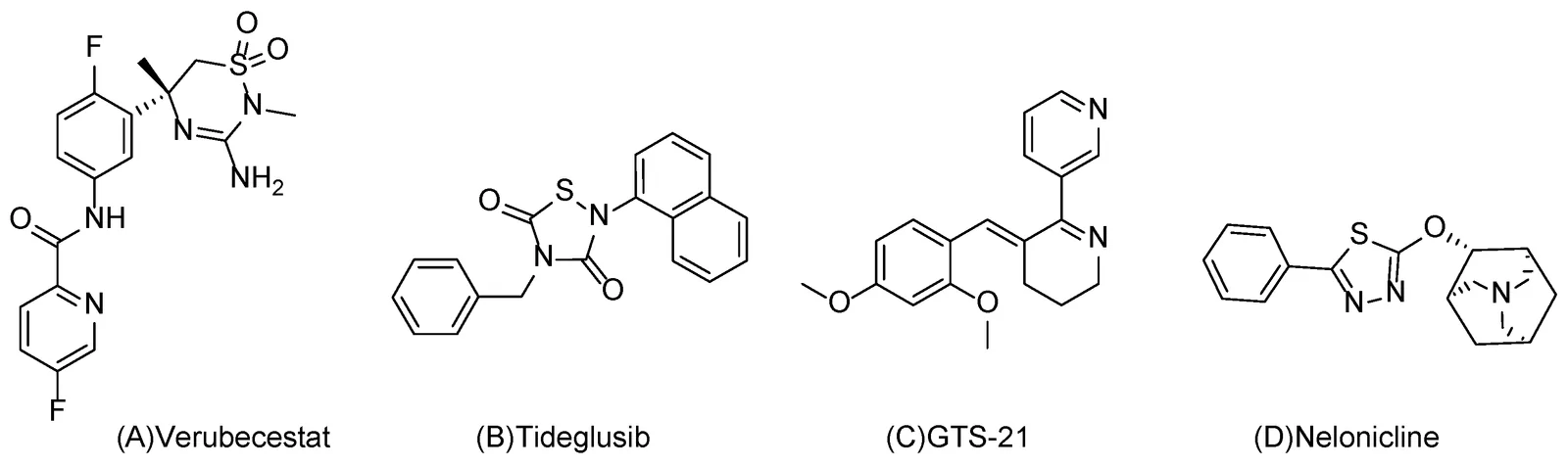
Representative synthetic small molecules with anti-Alzheimer’s activities.

Marine natural products exert anti-Aβ activity at four successive stages of the amyloid cascade. Stage 1: preventing the loss of native protein conformation. At the stage of Aβ monomers, certain marine polyphenols stabilize their native conformation and suppress protein misfolding, thereby blocking the cascade at an upstream level. This strategy aims to preserve the biologically active structure of proteins and represents one of the most effective prophylactic interventions. Stage 2: inhibiting the formation of amyloid fibrils. Misfolded Aβ monomers further assemble into oligomers and protofibrils. Marine natural products intervene at this stage through a dual pathway. First, they inhibit Aβ production by suppressing the enzymatic activities of β-secretase (BACE-1) and γ-secretase, thereby blocking the aberrant cleavage of amyloid precursor protein (APP) [20]. Marine alkaloids (e.g., manzamine derivatives) and brown-algae-derived polyphenols specifically inhibit BACE-1 and reduce the generation of Aβ_40_ and Aβ_42_, with an inhibitory efficacy comparable to that of clinical agents [21]. As a reference, the synthetic small-molecule **Verubecestat** (MK-8931, Figure 10A, Table 2), an orally bioavailable selective BACE-1 inhibitor, has advanced to Phase III clinical trials. Although it failed to achieve the cognitive improvement endpoint, it has provided critical clinical evidence for disease-modifying therapies targeting the amyloid pathway and established a reference framework for the development of marine-derived BACE-1 inhibitors [22]. Second, marine natural products directly impede the aberrant aggregation of Aβ. Marine polyphenols and terpenoids target the hydrophobic domains of Aβ monomers and oligomers, disrupt their β-sheet conformation, and thereby prevent the formation of fibrillar aggregates and senile plaques. Among them, red-algae-derived bromophenols specifically bind to Aβ_1–42_ monomers, markedly restrain oligomer formation, and mitigate subsequent neuronal toxicity. Stage 3: disrupting pre-formed amyloid fibrils. Certain marine polysaccharides can disassemble mature deposited Aβ fibrils to facilitate plaque clearance. This post-damage reparative strategy bears pathological relevance for patients with moderate-to-advanced AD [23]. In vitro lysozyme-based amyloid models demonstrate that anionic polysaccharides can disaggregate mature fibrils and promote protein renaturation via direct physical interactions, with their anti-amyloid activity governed by molecular flexibility and charge density [24,25]. Although these findings originate from non-Aβ systems, they provide mechanistic references for understanding the anti-fibrillogenic potential of marine polysaccharides. Nevertheless, direct evidence targeting Aβ fibrils is still lacking, and whether marine polysaccharides exert comparable renaturing effects on AD-associated aggregates remains to be verified. To date, few marine natural products have been reported to realize the function of Stage 4 (restoration of native conformation), which constitutes an important research gap.

### 2.2. Inhibition of Tau Protein Hyperphosphorylation

**Figure 4:**
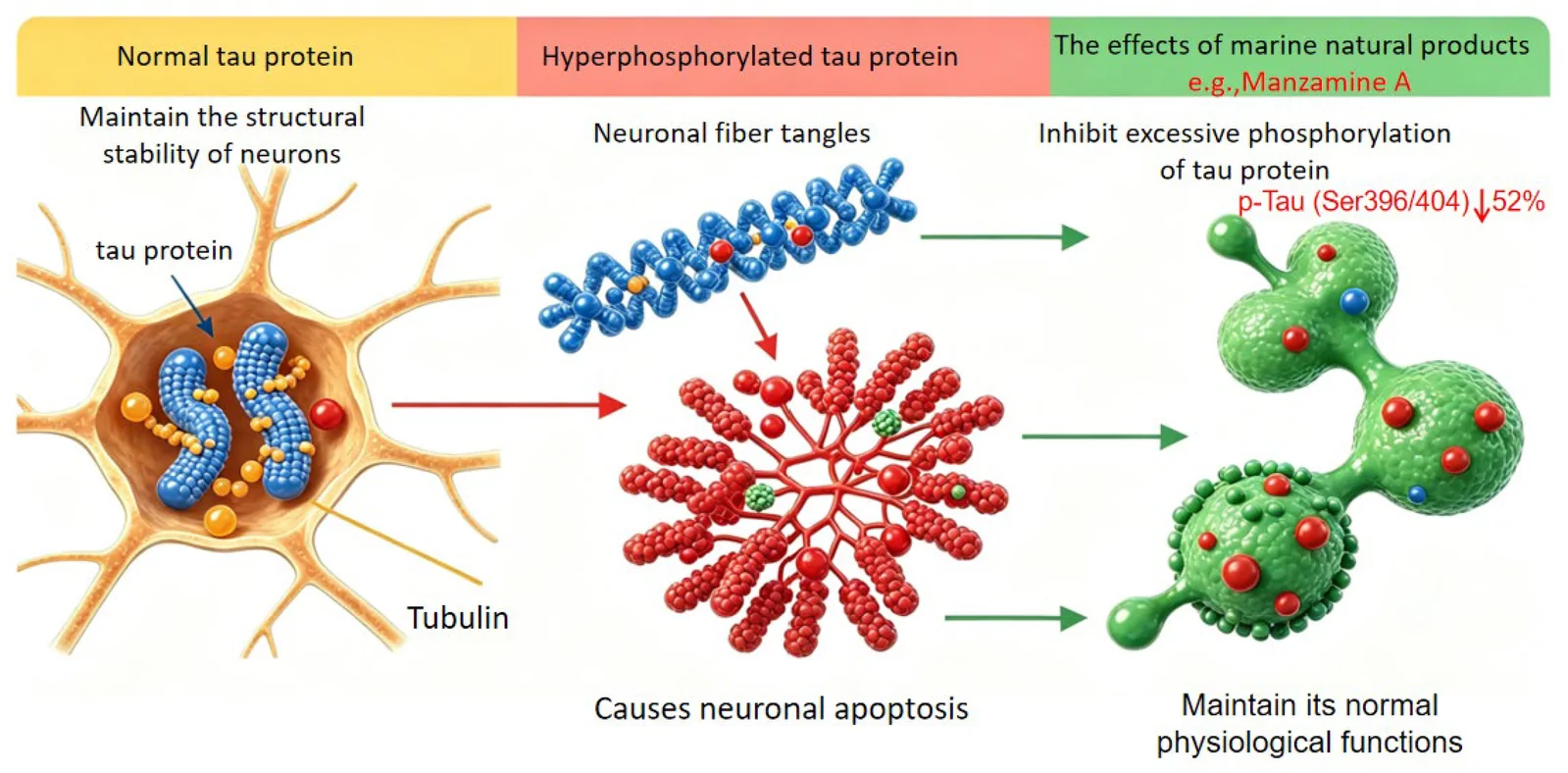
Schematic diagram of tau protein pathology and the intervention effects of marine natural products.

As a microtubule-associated protein, tau protein binds to microtubules under physiological conditions, stabilizes the microtubule architecture of neurons and mediates axonal transport [26]. During the pathological progression of AD, tau protein undergoes hyperphosphorylation and loses its capacity to bind microtubules. It subsequently aggregates to form neurofibrillary tangles [27], which disrupt the neuronal cytoskeleton, trigger axonal transport dysfunction and neuronal apoptosis, and ultimately lead to cognitive decline (Figure 4). Marine natural products can suppress tau hyperphosphorylation and maintain its normal physiological functions by regulating the activities of kinases and phosphatases involved in tau phosphorylation [28]. Furthermore, the representative small-molecule **Tideglusib** (NP-12, small-molecule) (Figure 10B, Table 2) is a classic irreversible GSK-3β inhibitor targeting tau pathology in AD and serves as a landmark compound for anti-Alzheimer’s disease research focusing on the tau pathway. It blocks tau hyperphosphorylation and neurofibrillary tangle formation, thereby alleviating neuronal injury [29,30]. It provides critical references for mechanistic exploration, efficacy optimization and the development of novel inhibitors of tau-targeted disease-modifying drugs for AD, and is a widely cited classic small molecule in studies of tau pathology.

### 2.3. Inhibition of Neuroinflammation and Regulation of Microglial Function

Neuroinflammation acts as a critical driver of AD progression and persists throughout the entire pathological process of AD. As resident immune cells in the central nervous system, microglia remain quiescent under physiological conditions and participate in neuronal trophic support and injury repair [31]. Upon stimulation such as Aβ deposition and tau aggregation, microglia become excessively activated and polarize into the pro-inflammatory M1 phenotype. They subsequently secrete abundant pro-inflammatory cytokines including TNF-α, IL-1β and IL-6, as well as nitric oxide (NO), which exacerbate neuronal damage and create a vicious cycle of inflammation and injury [32]. Marine natural products exert anti-inflammatory and neuroprotective effects by suppressing inflammatory pathways and modulating microglial polarization.

**Figure 5:**
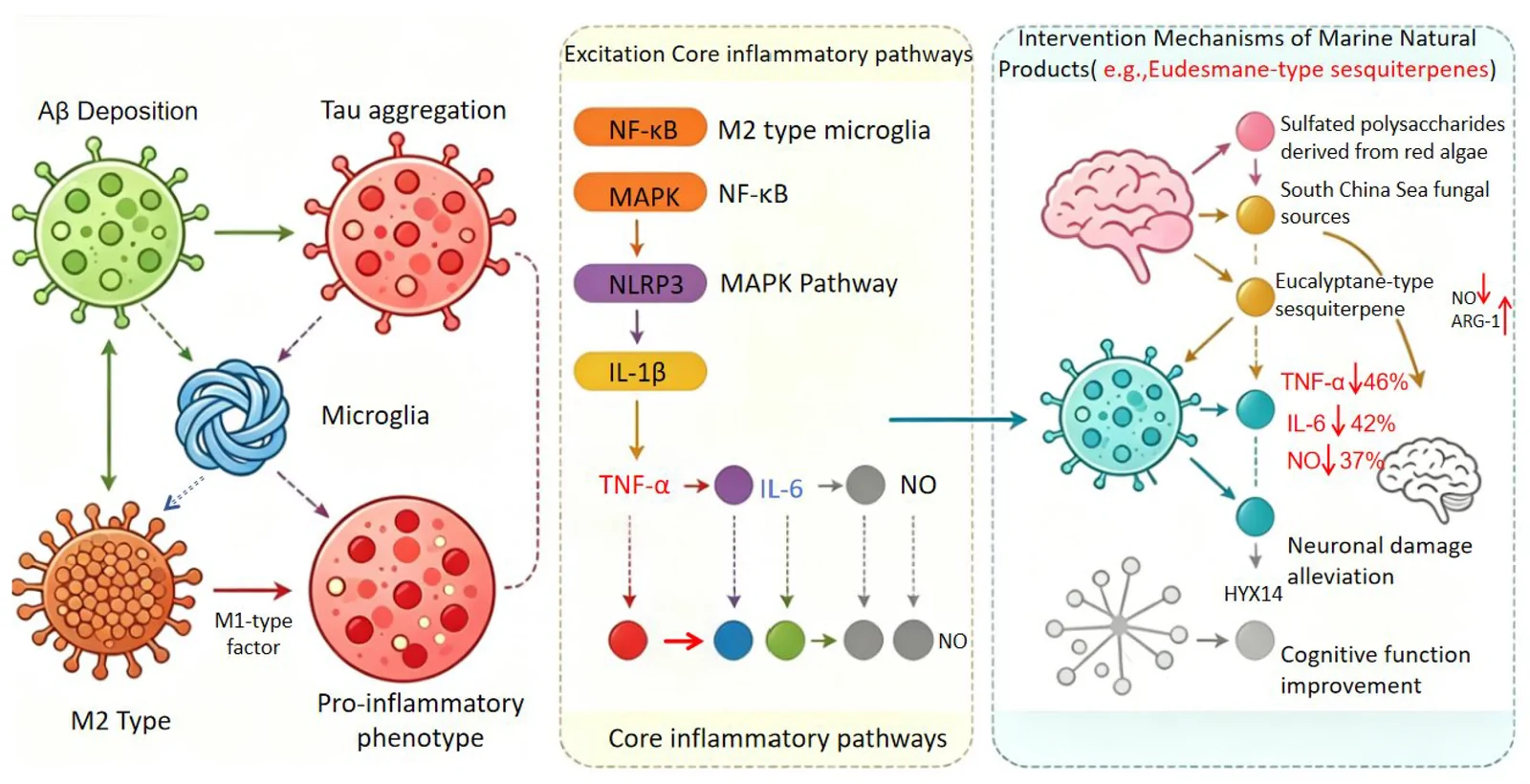
Neuroinflammatory mechanisms in AD and the intervention of marine natural products.

Nuclear factor κB (NF-κB), mitogen-activated protein kinase (MAPK) and NLRP3 inflammasome are the core pathways governing neuroinflammation. Marine natural products can reduce the release of pro-inflammatory cytokines by blocking the activation of these pathways (Figure 5) [33]. Eudesmane-type sesquiterpenes derived from marine fungi of the South China Sea downregulate the expression of inducible nitric oxide synthase (iNOS) and thereby decrease NO production. Meanwhile, this compound can upregulate the expression of arginase-1 (ARG-1), promote the polarization of microglia toward the anti-inflammatory M2 phenotype, and enhance its neuroprotective effects [34]. The representative compound GTS-21 (small molecule, see Figure 10C, Table 2), as validated by Tak’s research team at Kyoto Pharmaceutical University, can not only promote microglial phagocytosis of Aβ via the CaM-CaMKII/Rac1 signaling pathway but also inhibit γ-secretase activity to reduce Aβ production, thereby effectively ameliorating cognitive dysfunction in animal models [35].

### 2.4. Anti-Oxidative Stress and Mitochondrial Function Protection

**Figure 6:**
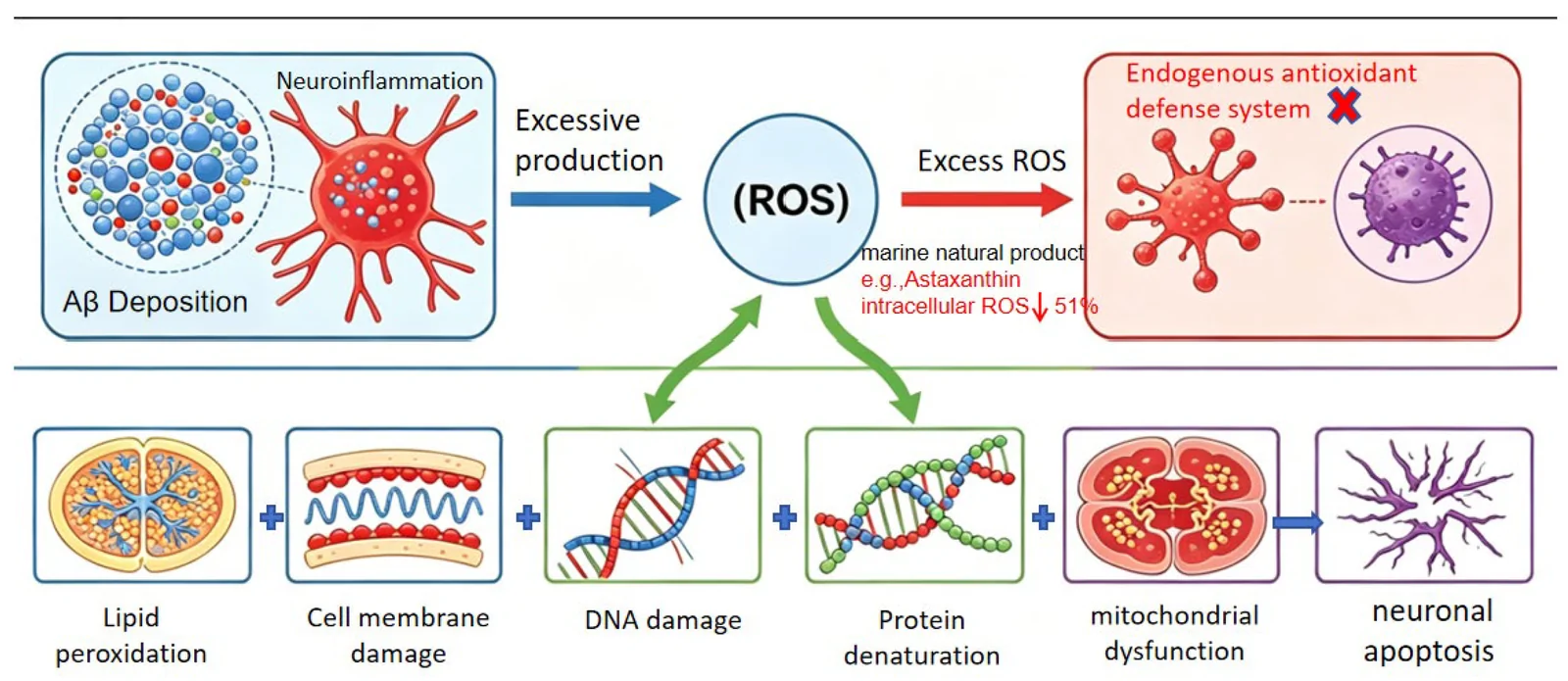
ROS-mediated oxidative damage pathway in AD.

Oxidative stress is an early event in the onset and progression of AD. Pathological processes including Aβ aggregation and neuroinflammation trigger excessive production of reactive oxygen species (ROS), which cannot be efficiently eliminated by the endogenous antioxidant system (Figure 6) [36]. This situation further causes neuronal lipid peroxidation, DNA damage and protein denaturation, impairs mitochondrial function, reduces mitochondrial membrane potential and disturbs energy metabolism, ultimately leading to neuronal apoptosis [37]. Many marine natural products possess potent antioxidant activity. They alleviate oxidative damage and preserve mitochondrial function via a dual mechanism of directly scavenging ROS and activating the endogenous antioxidant system.

Marine polyphenols (e.g., *brown algae* polyphenols and bromophenols), carotenoids (e.g., astaxanthin and fucoxanthin) and phycobiliproteins (e.g., phycoerythrin and phycocyanin) can directly scavenge reactive oxygen species such as superoxide anion and hydroxyl radical, reduce the production of lipid peroxidation products represented by malondialdehyde, and maintain the integrity of neuronal cell membranes [38]. Meanwhile, these compounds activate the nuclear factor erythroid 2-related factor 2 (Nrf2)/heme oxygenase-1 (HO-1) antioxidant pathway, upregulate the expression of antioxidant enzymes including superoxide dismutase (SOD) and glutathione peroxidase (GSH-Px), and thereby enhance the endogenous antioxidant defense capacity [39]. In addition, sea cucumber cerebrosides and marine steroids can reverse Aβ-induced decline in mitochondrial membrane potential, inhibit ROS overproduction, restore mitochondrial energy metabolism and suppress neuronal apoptosis [40].

### 2.5. Modulation of Cholinergic System Function

**Figure 7:**
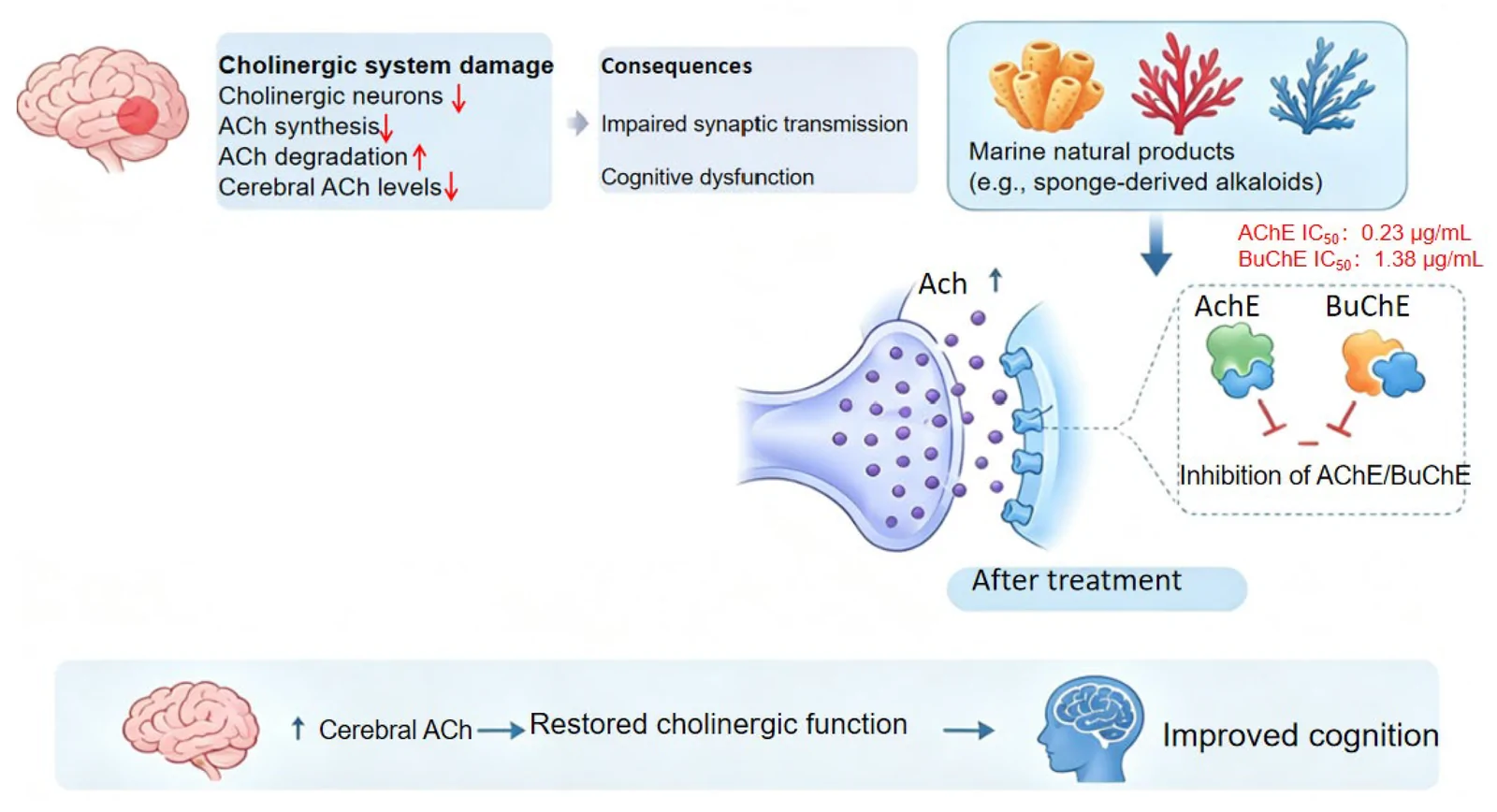
Cholinergic damage in Alzheimer’s disease and intervention by marine natural products.

Damage to the cholinergic system is a major contributor to cognitive dysfunction in AD. In the brains of AD patients, massive loss of cholinergic neurons occurs, accompanied by reduced synthesis and accelerated degradation of ACh. The resultant sharp decline in cerebral ACh levels disrupts synaptic transmission and eventually impairs learning and memory abilities [41]. Marine natural products can suppress the activities of AChE and BuChE, slow down ACh degradation, elevate cerebral ACh content, restore cholinergic function and ameliorate cognitive impairment (Figure 7).

Marine alkaloids (e.g., **saxitoxin** derivatives and sponge alkaloids), terpenoids and polyphenols exhibit potent AChE inhibitory activity, and some compounds show effects comparable to those of the clinical drug donepezil [42]. For instance, sponge-derived alkaloids competitively bind to the active site of AChE to block its catalytic activity and reduce ACh hydrolysis [43]. Carotenoids from *red algae*, such as lutein and zeaxanthin, concurrently inhibit AChE and BuChE to raise cerebral ACh levels. Meanwhile, they exert anti-inflammatory and antioxidant effects, achieving multi-target synergistic effects against AD [44]. Moreover, fucoidan improves cognitive function via AChE inhibition. It also interacts with muscarinic receptor 3 (M3R) to facilitate neurotransmitter release, further modulating the function of the cholinergic system [45].

### 2.6. Promotion of Neurotrophy and Synaptic Repair

**Figure 8:**
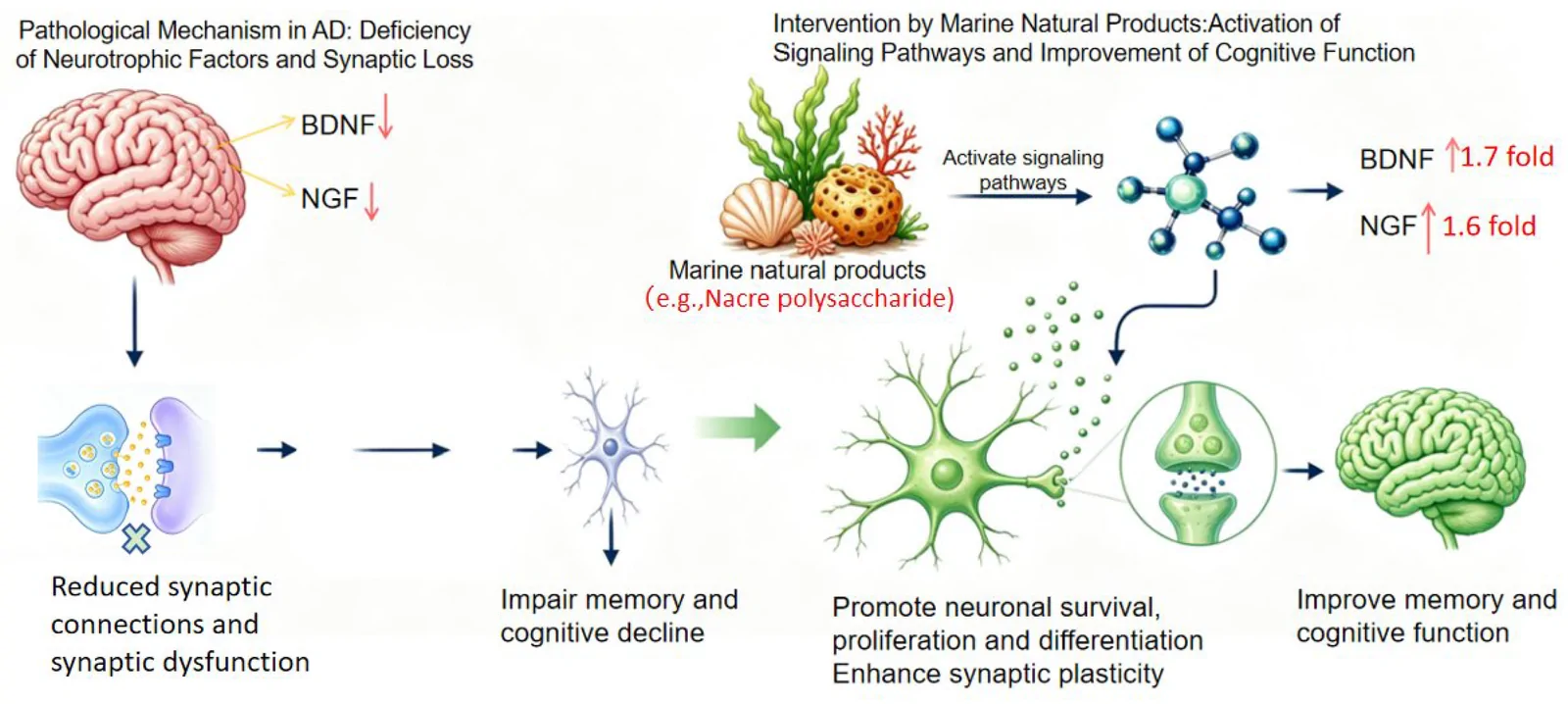
Neurotrophic factor dysregulation in AD and the intervention effects of marine natural products.

Deficiencies in neurotrophic factors (e.g., brain-derived neurotrophic factor, BDNF, and nerve growth factor, NGF) and synaptic loss are among the key mechanisms underlying cognitive decline in AD [46]. BDNF facilitates neuronal survival, proliferation and differentiation, enhances synaptic plasticity, and improves learning and memory performance [47]. In the brains of AD patients, BDNF levels decrease markedly, which leads to reduced synaptic connections and impaired synaptic function, and further exacerbates cognitive deterioration. By activating related signaling pathways, marine natural products can upregulate the expression of neurotrophic factors, repair synaptic damage and ameliorate cognitive function (Figure 8) [48,49]. In addition to marine natural products, artificially synthesized small molecules can also repair synaptic damage and ameliorate Alzheimer’s disease (AD) pathology. Nelonicline (ABT-126) (small molecule, Figure 10D, Table 2) is capable of crossing the blood–brain barrier to activate central α7 receptors, modulate cholinergic neurotransmission, remodel synaptic plasticity, and restore impaired nerve conduction function [50]. Meanwhile, it alleviates inflammation-mediated synaptic damage and neuronal apoptosis via the cholinergic anti-inflammatory pathway to exert multiple neuroprotective effects, making it a representative small molecule targeting synaptic repair.

### 2.7. Modulation of Gut–Brain Axis Function

**Figure 9:**
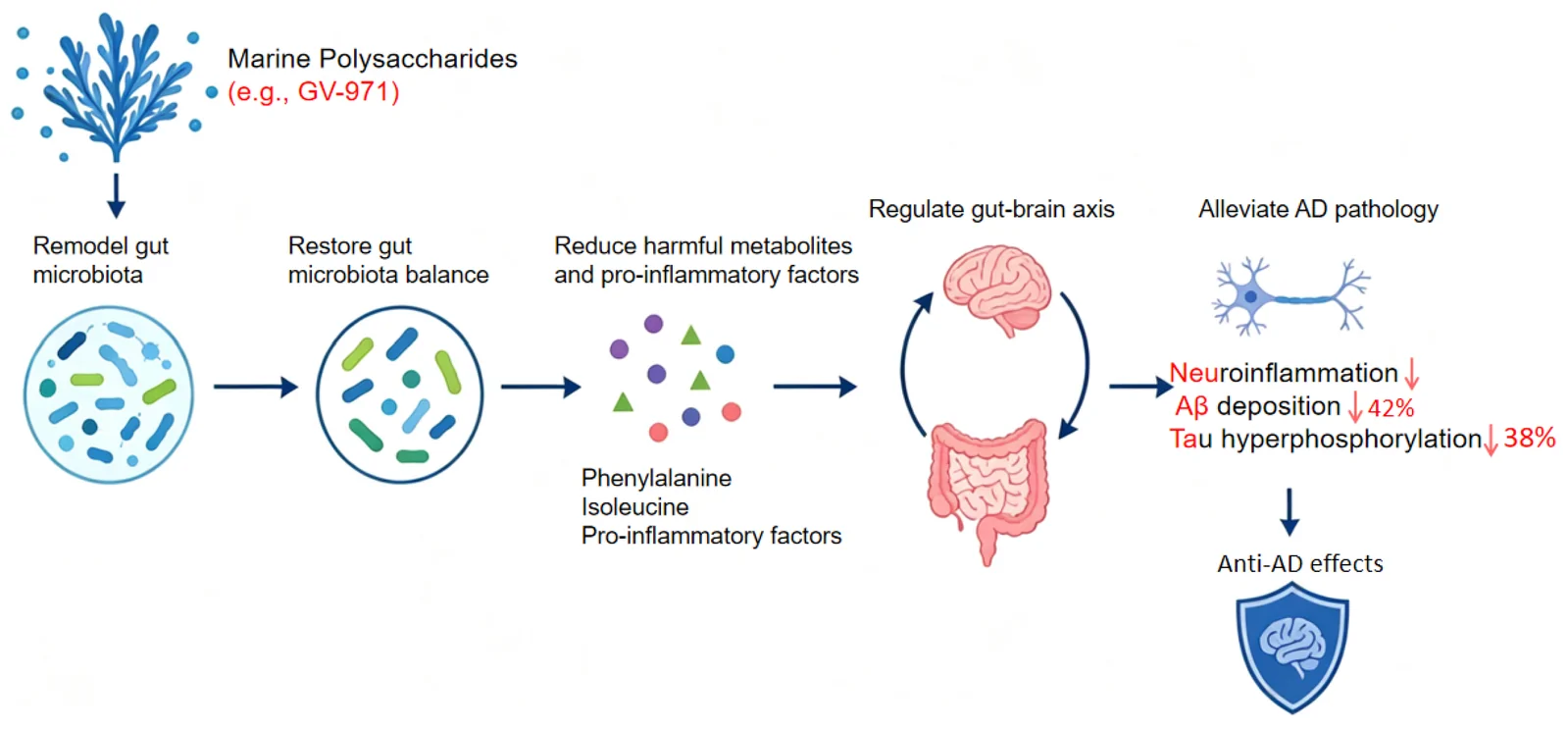
Gut–brain axis dysregulation in AD and intervention by marine natural products.

In recent years, the role of gut–brain axis dysfunction in the pathological progression of AD has attracted extensive research attention. Gut microbiota imbalance leads to increased levels of peripheral pro-inflammatory factors and accumulation of metabolic toxins such as phenylalanine and isoleucine [51]. These substances further affect the central nervous system via the gut–brain axis, thereby aggravating neuroinflammation, Aβ deposition and tau protein hyperphosphorylation [52]. Marine natural products, especially marine polysaccharides, can remodel the composition of gut microbiota and regulate gut–brain axis function, thus exerting anti-AD effects indirectly (Figure 9).

Owing to their unique advantages including novel chemical structures, diverse biological activities, low toxicity and high efficacy, marine natural products have been proven to be excellent sources of lead compounds for regulating the gut–brain axis and intervening in AD pathogenesis. Among them, marine polysaccharides, carotenoids and peptides are the most extensively investigated. They exert prominent anti-AD pharmacological activities through the linked mechanism of regulating gut microecology, repairing intestinal barrier, suppressing neuroinflammation and protecting neurons along the gut–brain axis [53,54]. This provides novel therapeutic targets and research strategies for AD prevention and the development of natural medicines.

## 3. Research Progress on Anti-AD Effects of Different Classes of Marine Natural Products

Marine natural products are characterized by abundant species and diverse chemical structures. Researchers have identified a large number of compounds with unique skeletons from marine environments, including polysaccharides, carotenoids [55], polyphenols, sterols and alkaloids, all of which possess prominent biological and pharmacological activities. In fact, numerous studies have demonstrated that marine natural products exert anti-inflammatory [56], antioxidant, anti-tumor and hypotensive effects [57]. To date, a variety of marine-derived agents have been approved for clinical application. For instance, plitidepsin (Aplidin), isolated from *Candida albicans*, is clinically used for the treatment of multiple myeloma [58]. Ziconotide, a non-opioid analgesic derived from the venom of Conus magus, is applied to relieve severe chronic neuropathic pain. Marine natural products have also exhibited remarkable therapeutic efficacy in the treatment of AD. As the first marine drug approved for AD intervention, GV-971 plays a pivotal role in bridging marine ecosystem resources and AD therapy [20]. Based on chemical structures, marine natural products with anti-AD potentials can be classified into polysaccharides, terpenoids, alkaloids, polyphenols, steroids and lipids. Compounds of different categories differ in anti-AD activities and action mechanisms. Here, we systematically review the research advances on representative marine natural products of each category against AD.

### 3.1. Marine Polysaccharides

Marine polysaccharides are high-molecular-weight polymeric carbohydrates composed of more than ten monosaccharide units linked via glycosidic bonds. According to their biological sources, they can be categorized into algal polysaccharides, marine animal polysaccharides and microbial polysaccharides. Among them, fucoidans take L-fucose and sulfate groups as the core structural units [59]. As one of the most extensively investigated marine natural products with the widest distribution, marine polysaccharides are mainly isolated from marine algae (*brown algae*, *red algae* and *green algae*) [60], marine animals (sea cucumbers, scallops and abalones), and marine microorganisms (bacteria and fungi). They possess favorable water solubility, low toxicity and multi-target pharmacological activities. Marine organisms such as algae and sponges have adapted to extreme marine environments and evolved distinctive metabolic pathways, rendering them valuable resources for the discovery and development of novel potent drugs [61]. Accordingly, marine polysaccharides stand out as one of the most promising candidates for anti-AD research.

**Figure 11:**
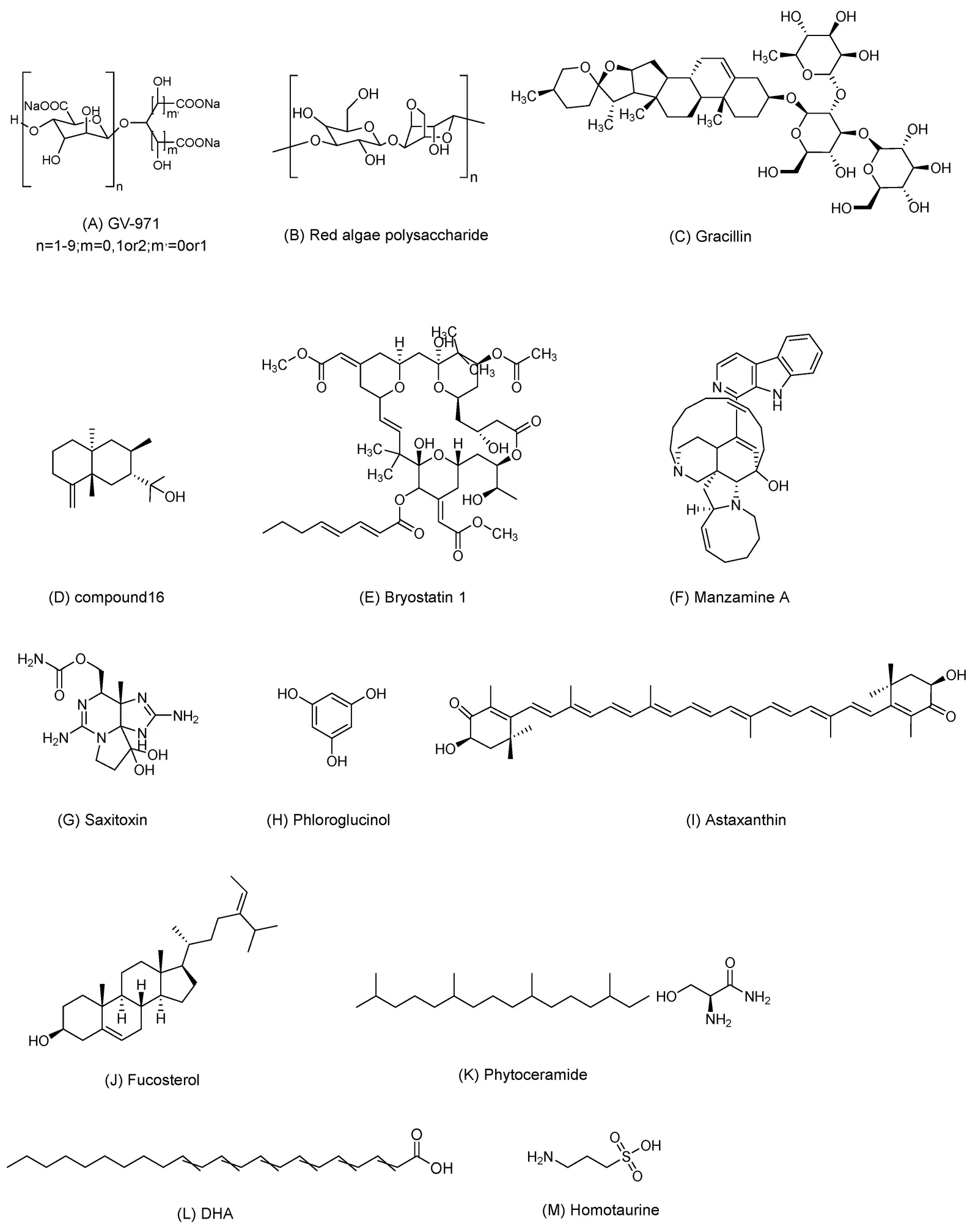
Marine natural molecular chemical structures with anti-Alzheimer’s disease properties.

As the predominant fraction of marine polysaccharides, algal polysaccharides, especially brown and red algal polysaccharides, have been most extensively investigated in the research of AD [62]. Brown algal polysaccharides, such as algal oligosaccharides and fucoidans, exert anti-AD activities via multiple mechanisms, including inhibiting Aβ aggregation, relieving inflammation, eliminating oxidative stress and regulating the gut–brain axis [63]. Brown algal oligosaccharides can markedly improve cognitive function in mice with Aβ-induced AD, reduce cerebral Aβ deposition and tau protein phosphorylation, and suppress neuroinflammation and oxidative stress [64]. Developed by Geng’s team at the Shanghai Institute of Materia Medica, Chinese Academy of Sciences, sodium oligomannate (GV-971, Jiuqi-1) (Figure 11A) is the world’s first anti-AD drug targeting the gut–brain axis [65]. Derived from brown algal oligosaccharides, it remodels gut microbiota composition, reduces the accumulation of metabolic toxins, and prevents peripheral inflammatory factors from infiltrating the brain. These effects collectively alleviate cerebral neuroinflammation and inhibit the deposition of Aβ and tau proteins. Its Phase III clinical trial showed that after 36 weeks of continuous administration, patients with mild-to-moderate AD achieved a 2.54-point improvement in ADAS-Cog12 scores relative to the placebo group, indicating a significant enhancement in cognitive function [66]. Yu’s group at Ocean University of China isolated sulfated fucoidans from *brown algae*. Studies using APP/PS1 mouse models confirmed that these polysaccharides activate the Nrf2/HO-1 antioxidant pathway, attenuate oxidative stress, suppress Aβ deposition and tau protein hyperphosphorylation, and markedly improve learning and memory performance in model mice [67].

Red algal polysaccharides (Figure 11B) possess a typical linear galactan structure, which consists of alternating β-D-galactose and α-L-galactose/3,6-anhydro-L-galactose residues. Sulfation commonly occurs at the C-4 and C-6 positions, giving rise to different carrageenan subtypes, including κ-, ι- and λ-carrageenan. The density and location of sulfate groups directly govern their anti-inflammatory and antioxidant capacities [68]. Sulfate groups also improve the water solubility of polysaccharides and facilitate the formation of unique three-dimensional conformations, enabling specific binding to diverse cellular receptors. Such polysaccharides exert anti-AD effects mainly via three principal pathways: inhibition of NF-κB-mediated neuroinflammation, upregulation of BDNF expression and modulation of mitochondrial function to mitigate neuronal damage, as well as activation of the Nrf2/HO-1 antioxidant pathway to eliminate ROS and alleviate oxidative stress injury [69]. Accumulated studies have verified that sulfated red algal polysaccharides markedly activate the Nrf2/HO-1 pathway in the SH-SY5Y cell model. Specifically, the expression level of HO-1 is upregulated by 2.8-fold, the intracellular ROS content is reduced by 59.3%, and cell viability increases to 82.7%. These changes endow the polysaccharides with potent antioxidant and neuroprotective effects, thereby ameliorating cognitive function [70]. Furthermore, red algal polysaccharides exhibit prominent anti-inflammatory and anti-apoptotic activities in LPS-stimulated BV2 microglia and Aβ_1–42_-injured PC12 cell models. They effectively suppress the release of pro-inflammatory cytokines and significantly downregulate the levels of tumor necrosis factor-α (TNF-α), interleukin-1β (IL-1β) and interleukin-6 (IL-6) while reducing NO production to block the NF-κB-triggered inflammatory cascade. In terms of cell survival, these polysaccharides remarkably decrease the Aβ-induced neuronal apoptosis rate from 38.5% to 12.3%, maintain mitochondrial membrane potential, and elevate the expression of BDNF by 2.1-fold, ultimately optimizing the overall neuronal microenvironment [59,67].

Marine animal polysaccharides also exert prominent anti-AD activities. Polysaccharides derived from sea cucumber, scallop and abalone can eliminate reactive ROS, inhibit Aβ aggregation, and alleviate Aβ-induced cognitive impairment. Sea cucumber polysaccharides suppress AChE activity to elevate the level of ACh in the brain, and inhibit neuroinflammation to protect neurons [71]. Scallop polysaccharides activate the Nrf2/HO-1 pathway, enhance antioxidant capacity, reduce Aβ deposition and tau phosphorylation, and improve learning and memory functions in AD model animals [72].

### 3.2. Marine Terpenoids

Marine terpenoids are built up with isoprene units (C5) as the fundamental structural unit. According to the number of isoprene units, they are classified into monoterpenes (C10), sesquiterpenes (C15) diterpenes (C20) and triterpenes (C30), with carbon skeletons including acyclic, monocyclic, bicyclic, tricyclic and tetracyclic frameworks [73]. Marine diterpenes and sesquiterpenes are predominantly biosynthesized under the catalysis of terpene synthases, and further undergo cytochrome P450-mediated oxidation, cyclization and rearrangement to form complex fused-ring structures [74]. Terpenoids isolated from sponges generally undergo halogenation, lactonization and skeletal rearrangement. These modifications endow them with higher structural stability and lipophilicity, enabling easier penetration across the blood–brain barrier.

Among diterpenoid compounds, gracilin (small molecule, Figure 11C) acts as an antioxidant via mitochondrial targeting and induction of Nrf2 translocation [75]. The research group led by Marta Leirós investigated the effects and underlying mechanisms of gracilins against AD using combined neuroblastoma cell models (BE(2)-M17, SH-SY5Y-TMHT441) and in vivo 3 × Tg-AD mouse models. The experimental results demonstrated that gracilins possess potent anti-AD activities. They exert multifunctional effects by inhibiting β-site amyloid precursor protein cleaving enzyme 1 (BACE-1) and extracellular signal-regulated kinase (ERK) activities, as well as suppressing tau protein hyperphosphorylation, all of which are critical pathogenic processes of AD. In in vivo assays, these compounds tended to improve learning ability and spatial memory in mice. With multi-target anti-AD properties, gracilins are promising candidate compounds for anti-AD drug development [76].

Huang’s group isolated eudesmane-type sesquiterpenes from the marine fungus *Eutypella* sp. collected from the South China Sea. In vitro experiments using an LPS-induced inflammatory model of BV2 microglia revealed that these compounds could inhibit the release of pro-inflammatory cytokines and reverse the pro-inflammatory polarization of microglia, with no obvious cytotoxicity. After intragastric administration for 8 weeks to APP/PS1 double-transgenic AD mice in vivo, the compounds markedly alleviated cognitive deficits, relieved cerebral inflammation and reduced neuronal damage in the hippocampus. A total of 23 eudesmane-type sesquiterpenes were isolated and identified from the strain *E.* sp. F0219. Among them, Compound 16 (small molecule, Figure 11D) significantly downregulated the expression of inflammatory factors including IL-6 and TNF-α, decreased the level of iNOS and upregulated ARG-1. It further suppressed NF-κB activation-mediated M1 polarization of microglia, thereby exerting anti-neuroinflammatory effects [77]. In addition, the research team designed and synthesized an entatisane-type diterpenoid designated HXY14. In vitro assays indicated that HXY14 could specifically bind to and inhibit soluble epoxide hydrolase (sEH), exerting anti-inflammatory and neuroprotective effects. After 6 weeks of intragastric administration in AD model mice, HXY14 alleviated neuroinflammation and improved cognitive function via suppressing the sEH pathway with favorable safety profiles. HXY14 serves as a novel lead compound for the treatment of AD.

### 3.3. Macrolides

Marine macrolides are biosynthesized by polyketide synthases (PKSs), which catalyze the elongation and cyclization of acyl chains bound to acyl carrier proteins. They feature a core skeleton consisting of 16- to 40-membered lactone rings decorated with various substituents such as hydroxyl, keto, methyl, epoxy and glycosyl groups. The bryostatin family possesses an 18-membered lactone ring as the parent structure, comprising three ring systems designated A, B and C. The unsaturated lactone bond between C2 and C7 and the ester side chain at the C20 position serve as the key structural domains for binding to PKCε [78].

Possessing unique structures and versatile biological activities, macrolides exhibit remarkable potential, especially in neuroprotection, anti-inflammation and inhibition of amyloid aggregation. As a typical member, bryostatin-1 (macromolecule, Figure 11E) is a highly oxidized marine macrolide initially isolated from the marine bryozoan *Bugula neritina*. To date, more than 20 family members (bryostatin-1 to bryostatin-20) have been identified [79]. Compounds of this family show extremely high binding affinity to protein kinase C (PKC), with *K_i_* values ranging from 0.8 to 6 nM. They effectively regulate the PKC signaling pathway, thereby modulating synaptic plasticity and exerting neuroprotective effects. By activating PKCε, bryostatin-1 promotes the metabolism of amyloid precursor protein (APP) via the α-secretase pathway and reduces Aβ production. Meanwhile, it upregulates BDNF expression to facilitate synaptic regeneration and ameliorate cognitive function in AD model animals [79,80]. Currently, bryostatin-1 has progressed to Phase II clinical trials for AD treatment. Intravenous administration at a dose of 20 μg/m^2^ once every two weeks for 12 consecutive cycles yielded a 3.1-point improvement in patients’ ADAS-Cog scores and a 47.3% increase in BDNF levels. In cellular assays, 1 nmol/L bryostatin-1 activated PKCε and redirected APP processing toward the α-secretase pathway, leading to a 53.7% reduction in the production of Aβ_40_/Aβ_42_. These findings demonstrate its promising prospects for clinical application [81].

### 3.4. Marine Alkaloids

Marine alkaloids are nitrogen-containing basic compounds with nitrogen heterocycles as their core structures. They are mainly derived from sponges, corals, marine animals and marine microorganisms, and display diverse structural types, including indoles, pyridines, guanidines, β-carbolines, macrocyclic alkaloids and steroidal alkaloids [82]. Alkaloid subgroups differ distinctly in biosynthetic pathways and skeletal features. Manzamine alkaloids are characterized by intricate fused nitrogen heterocycles, which are biosynthesized via cyclization, oxidation and coupling of tryptophan and isoprene units [83]. Indole alkaloids originate from tryptophan precursors through oxidation, cyclization and bromination reactions [84]. **Saxitoxin** is classified as a guanidine alkaloid, possessing highly polar guanidino groups and hydrogenated purine ring systems [85]. These compounds exert prominent activities on the central nervous system and serve as valuable natural resources for the development of AD drugs. Most of them possess multi-target anti-AD properties [86]. The research team led by König at the Max Planck Institute in Germany conducted a series of experiments, including in vitro cholinesterase inhibition assays, neuronal damage protection tests, anti-inflammatory assessments in microglia, and behavioral and in vivo pharmacodynamic evaluations using AD model mice. The results revealed that manzamine A (small molecule, Figure 11F) markedly inhibited tau protein hyperphosphorylation in neurons and reduced the release of neuroinflammatory cytokines, raising cell viability by more than 45% [87]. Derivatives of **saxitoxin** (small molecule, Figure 11G) investigated in this study exhibited selective inhibitory activity against AChE with an IC_50_ value of approximately 2.1 μM, comparable to that of donepezil. These derivatives significantly elevated cerebral acetylcholine levels and effectively enhanced the learning and memory capabilities in AD model mice. Notably, their cytotoxicity and in vivo animal toxicity were only approximately 0.05 times that of natural **saxitoxin**, conferring excellent safety profiles [88]. With novel chemical architectures and potent biological activities, marine alkaloids can be further optimized for druggability via structural modification. They offer abundant privileged scaffolds for the research and development of novel anti-AD drugs.

### 3.5. Marine Polyphenols and Carotenoids

Marine polyphenols and carotenoids are two classes of compounds with remarkable antioxidant properties [89]. These compounds are mainly derived from marine algae, as well as crustaceans and shellfish like shrimps and crabs. They are characterized by low toxicity and excellent biocompatibility. Consequently, they are highly promising as crucial resources and potential lead compounds for dietary intervention and drug discovery in the fight against AD.

Marine polyphenols utilize phloroglucinol as their basic structural unit, primarily including phlorotannins derived from *brown algae* and bromophenols from *red algae*. Brown algal polyphenols are formed through C-C and C-O-C linkages [25]. They can inhibit Aβ aggregation and tau protein phosphorylation, activate the Nrf2/HO-1 antioxidant cascade, and alleviate oxidative stress and neuroinflammation. Red algal bromophenols, which possess benzene rings and bromine substituents, scavenge reactive ROS, impede Aβ assembly, exert anti-inflammatory effects, and inhibit acetylcholinesterase activity, thus providing neuroprotection through synergistic multi-target actions. Moreover, red algal polyphenols regulate the dopaminergic and Nrf2 pathways to improve AD-associated cognitive deficits [90]. Among these compounds, bromophenols have been investigated most comprehensively for their anti-AD potential.

The research team led by Ana M. M. Gonçalves from the University of Coimbra, Portugal, investigated phlorotannins (small molecule, Figure 11H) derived from brown and *red algae* using PC12 neuronal cell models to evaluate their protective effects against oxidative stress and Aβ-induced damage. The results demonstrated that treatment with 10 μmol/L phlorotannins PFFA and 974-A restored the viability of PC12 cells injured by 150–200 μmol/L tert-butyl hydroperoxide (t-BHP) from 59–63% to 81–94% [91]. In the H_2_O_2_-induced injury model, pretreatment with these polyphenols reduced intracellular ROS levels by 40–60% and decreased malondialdehyde (MDA) content by approximately 50%. Mechanistic studies revealed that these compounds enhanced the nuclear translocation of Nrf2 by 2–3 folds, and correspondingly upregulated the expression of downstream proteins: HO-1 by 1.8–2.5 folds, superoxide dismutase (SOD) by 1.5 folds, and glutathione peroxidase (GSH-Px) by 1.6 folds. Mitochondrial function assays indicated that the polyphenols recovered around 70% of the Aβ-induced decline in mitochondrial membrane potential and cut ROS leakage by nearly 50%. Meanwhile, they suppressed caspase-3 activation by 45%, leading to a marked reduction in neuronal apoptosis [92].

Carotenoids feature a conjugated polyene skeleton composed of eight isoprene units, with cyclization at both termini to form β-ionone rings. Their antioxidant capacity and lipophilicity are governed by the length of conjugated double bonds and terminal ring structures [93]. Capable of penetrating the blood–brain barrier (BBB), carotenoids scavenge free radicals, particularly singlet oxygen, thereby preventing extensive lipid peroxidation in the brain and conferring potential neuroprotective effects [93]. The research team led by Arunachalam Muthuraman at AIMST University, Malaysia, investigated the efficacy of astaxanthin (small molecule, Figure 11I) against cerebrovascular-associated AD [94], using SH-SY5Y neuronal cells and in vivo zebrafish AD models for functional verification. In cellular experiments, 10 μmol/L astaxanthin was applied to Aβ-damaged SH-SY5Y cells. The results showed that this compound suppressed Aβ aggregation, inhibited matrix metalloproteinase-13 (MMP-13) activity, and reduced neuronal apoptosis by around 40%. In the in vivo study, a zebrafish AD model with cerebrovascular impairment was established via streptozotocin (STZ) induction. After 21 consecutive days of treatment with astaxanthin at concentrations of 10–20 mg/L, behavioral tests including the light–dark box test and T-maze test demonstrated that astaxanthin relieved cognitive dysfunction in model organisms. It also maintained BBB integrity, attenuated cerebral Aβ deposition and downregulated MMP-13 expression, thereby markedly ameliorating the pathological characteristics of cerebrovascular-related AD. This work provides a new strategy targeting cerebrovascular-associated AD by modulating MMP-13 activity and preserving blood–brain barrier integrity for anti-AD therapeutic discovery.

### 3.6. Marine Steroids, Cerebrosides, Polyunsaturated Fatty Acids and Aminosulfonates

Marine steroids possess a tetracyclic cyclopentanoperhydrophenanthrene skeleton. Their biological activities are determined by the length and substituents of the side chain at the C17 position, while a hydroxyl group at C3 and a C5-C6 double bond represent their common structural features. These compounds are predominantly isolated from sponges and corals and exhibit considerable structural diversity. Steroids derived from sponges can specifically bind to the active site of AChE and inhibit its activity, thereby elevating cerebral ACh levels. Meanwhile, they exert antioxidant and anti-inflammatory effects, achieving multi-target synergistic effects against AD [95]. In 2018, a research team led by Siew Moi Phang from the University of Malaya, Malaysia, carried out a study on fucosterol (small molecule, Figure 11J). The Ellman method was employed to evaluate the in vitro inhibitory effects on AChE and BuChE. Simultaneously, inflammatory models of RAW264.7 and BV2 microglia induced by lipopolysaccharide (LPS) and amyloid–beta (Aβ) were established to systematically detect cholinesterase activity, the release of inflammatory factors such as TNF-α, IL-1β, IL-6, NO, and PGE2, and mRNA expression of inducible nitric oxide synthase (iNOS). The results indicated that the IC_50_ values of fucosterol against AChE and BuChE were 12.4 μM and 18.7 μM, respectively, suggesting a weak selectivity towards cholinesterases. At a concentration of 10 μM, fucosterol reduced the secretion of TNF-α, IL-1β, and IL-6 by 68%, 72%, and 59%, respectively. It also significantly decreased the levels of NO and PGE2 by 50–70% and downregulated iNOS mRNA expression by 80% [96]. These findings confirm that fucosterol exerts dual effects of cholinesterase inhibition and anti-neuroinflammation, presenting significant potential for the development of multi-target agents against AD.

Sea cucumber cerebrosides are significant bioactive components of sea cucumbers. They can be metabolized into ceramides to maintain cerebral function [97] and mitigate Aβ-induced neuronal damage and cognitive impairment. A collaborative research team led by Jong Hoon Ryu from Kyung Hee University and Ewha Woman’s University in South Korea explored the anti-AD effects of phytoceramide (small molecule, Figure 11K). In vitro models of PC12 cells and primary cortical neurons damaged by glutamate and Aβ_25–35_ were established to assess cell viability, apoptosis rate, and intracellular reactive ROS levels. In vivo mouse models of scopolamine-induced memory impairment and Aβ-injected Alzheimer’s disease were constructed. Cognitive functions were evaluated through the Morris water maze and passive avoidance tests, and pathological indicators and signaling pathways in brain tissues were further analyzed [98]. The findings indicated that phytoceramide enhanced the viability of damaged neurons by 40–50% and inhibited neuronal apoptosis, ROS accumulation, and abnormal tau phosphorylation. In animal experiments, it shortened the escape latency in the water maze by more than 30%. Simultaneously, it decreased Aβ plaque burden, upregulated BDNF expression, and restored cholinergic function, with no evident acute toxicity detected. These data validate its potent neuroprotective and cognition-improving properties. By suppressing oxidative stress and neuroinflammation, sea cucumber cerebrosides reduce Aβ deposition and tau hyperphosphorylation to safeguard neurons. They also promote the expression of neurotrophic factors, repair synaptic damage, and improve learning and memory abilities in AD model animals.

Polyunsaturated fatty acids, exemplified by docosahexaenoic acid (DHA), are predominantly sourced from marine fish and algae. As essential agents for nutritional intervention against AD, they exert therapeutic effects via modulating neuronal membrane fluidity, mitigating inflammation and promoting neurogenesis. DHA (small molecule, Figure 11L) can traverse the blood–brain barrier and incorporate into neuronal membranes to sustain their structural stability. It also suppresses Aβ aggregation and neuroinflammation, elevates BDNF expression, and ultimately ameliorates cognitive function [99,100]. Animal studies have demonstrated that DHA can reduce Aβ deposition, inhibit tau protein phosphorylation, and alleviate neurofibrillary pathology [101,102]. Some studies suggest that DHA decreases γ-secretase and BACE-1 activity by shifting the APP cleavage pathway toward non-amyloidogenic pathways, thereby promoting Aβ reduction [103,104]. Moreover, DHA acts synergistically with marine natural products such as astaxanthin to further strengthen anti-AD activity, indicating promising prospects for combined use. Metabolic pathway engineering of microalgae based on synthetic aminosulfonates are sulfur-containing compounds generated through the substitution of the hydroxyl groups of sulfuric acid molecules. **Homotaurine**, which has been isolated from diverse marine *red algae*, is biosynthesized from cysteine via three consecutive reactions: oxidation, decarboxylation, and sulfonation. Possessing both basic amino groups and acidic sulfonic acid groups, it exists in the form of a zwitterion, characterized by excellent water solubility, and serves as a typical natural aminosulfonate [105]. Clinical research has indicated that **homotaurine** (small molecule, Figure 11M) exerts neuroprotective effects through dual mechanisms. On one hand, it encapsulates amyloid peptides to impede their misfolding and aggregation, and obstructs the formation of neurotoxic species such as Aβ oligomers, thereby alleviating neuronal damage [54]. As crucial pathogenic factors for neurological disorders, these oligomers further assemble into Aβ protofibrils and fibrils, ultimately resulting in amyloid plaque deposition. Phase II clinical trials carried out by the research team led by Hartmann at Grenoble Alpes University, France, disclosed that the daily administration of 1500 mg of **homotaurine** reduced cerebrospinal fluid Aβ_42_ levels by 19.7% in patients with mild-to-moderate AD. In vitro assays demonstrated that 5 mmol/L of homotaurine inhibited Aβ aggregation by 63.2% [106], with a favorable safety profile, suggesting its therapeutic potential. However, the subsequent Phase III ALPHASE trial targeting mild-to-moderate AD failed to achieve the primary efficacy endpoints. Although patients showed a tendency of improvement in the total score of the Clinical Dementia Rating and statistically significant differences were observed in the Alzheimer’s Disease Assessment Scale-Cognitive subscale, pre-specified analyses of psychometric data revealed no remarkable statistical differences [107]. Notably, the secondary endpoints of this trial still verified that homotaurine exerts neuroprotection by suppressing Aβ activity. Therefore, further investigations are necessary to clarify its therapeutic efficacy against AD.

### 3.7. Summary

This section provides a narrative overview of the research progress on representative marine natural products from each chemical class against AD. The detailed experimental parameters—covering compound identities, in vitro and in vivo models, dosages, administration routes, treatment durations, proposed mechanisms, and references—are compiled in Table 1 to facilitate rapid cross-comparison.

**Table 1:** Summary of experimental evidence for anti-AD marine natural product.

Compound	Source	Experimental Model	Dose/Concentration	Administration Route	Treatment Duration	Mechanism of Action	Ref.
Manzamine alkaloids	Marine sponge (Acanthostrongylophora ingens, Haliclona sp.)	SH-SY5Y cell; BV2 microglia	1–10 μM	in vitro	24–48 h	Inhibit BACE-1 activity, suppress neuroinflammation	[79]
Fucoidan	Marine *brown algae*	APP/PS1 mice	100 mg/kg	intragastric	8 weeks	Inhibit Aβ aggregation; activate Nrf2/HO-1 pathway	[59]
Eudesmane-type sesquiterpenes (Compound 16)	Marine-derived fungus E. sp. F0219	BV2 microglia; APP/PS1 mice	5 μM (cell); 20 mg/kg (mice)	in vitro incubation; intragastric	24 h (cell); 8 weeks (mice)	Inhibit NF-κB, promote M2 microglia polarization	[76]
Gracilin	Marine sponge Spongionella gracilis	BE(2)-M17, SH-SY5Y-TMHT441; 3 × Tg-AD mice	0.1–5 μM; 12 mg/kg	in vitro; intraperitoneal	48 h; 12 weeks	Inhibit BACE-1 and ERK; reduce tau hyperphosphorylation	[69]
Bryostatin-1	Marine bryozoan B. neritina	Multiple neuronal cell; AD patients	1 nmol/L (cell); 20 μg/m^2^ (human)	in vitro; intravenous	24 h; every two weeks, 12 cycles	Activate PKCε; up-regulate BDNF	[73]
Astaxanthin	Marine microalgae, crustaceans and salmon	SH-SY5Y STZ-induced zebrafish AD model	10 μM; 10–20 mg/L (water-borne)	in vitro; water immersion	24 h; 21 days	Inhibit MMP-13; maintain BBB integrity	[88]
Fucosterol	Marine *brown algae*	RAW264.7, BV2 microglia	10 μM	in vitro incubation	24 h	Inhibit AChE/BuChE; suppress pro-inflammatory factors	[25]
Homotaurine	Marine *red algae*	mild-moderate AD patients	1500 mg/day (human)	oral	clinical continuous administration	Block Aβ misfolding and aggregation	[99]
Sponge-derived alkaloids (Aaptamine)	Marine sponge Aaptos aaptos/Aaptos suberitoides	In vitro: neuronal cell models	IC_50_: 0.23 μg/mL	in vitro incubation	—	Competitively binds AChE active site; blocks catalytic activity and ACh hydrolysis	[42]
GV-971	Extract of marine *brown algae* (oligomannate)	Human, mild-to-moderate AD patients	900 mg/day	oral	36 weeks	Remodels gut microbiota, reduces metabolic toxic metabolites, relieves cerebral neuroinflammation, inhibits Aβ and tau protein deposition	[64,65]
Phlorotannins (PFFA,974-A)	Marine brown algae (Ecklonia sp.)	In vitro: PC12 neuronal cells	10 μmol/L	in vitro incubation	24 h	Activates Nrf2/HO-1 antioxidant signaling; restores mitochondrial function; suppresses neuronal apoptosis	[90,91]
Phytoceramide	Marine sponge (Monanchora clathrata, Mycale euplectellioides)	In vitro: PC12, primary cortical neurons In vivo: scopolamine-/Aβ-challenged AD mice	In vivo: 10, 25, 50 mg/kg In vitro:—	in vitro incubation; oral (p.o.)	7 days (Morris water maze); 13 days (passive avoidance test)	Inhibits neuronal apoptosis, ROS accumulation and abnormal tau hyperphosphorylation; up-regulates BDNF; restores cholinergic function	[97]
DHA	marine fish oil	In vitro: neuronal cells; In vivo: AD model mice	400 mg/kg·bw/10 μmol/L	in vitro incubation; oral/intragastric	In vivo: 8 weeks; In vitro: 24 h	Inhibits Aβ aggregation and tau hyperphosphorylation; elevates BDNF level; modulates neuronal membrane fluidity	[98,99,100,101,102,103]
Sulfated fucoidans	Marine *brown algae*	APP/PS1 mouse model	—	Intragastric	—	Activate Nrf2/HO-1 pathway; attenuate oxidative stress; suppress Aβ deposition and tau phosphorylation	[66]
Brown algal oligosaccharides	Marine *brown algae*	Aβ-induced AD mice	—	—	—	Improve cognition; reduce Aβ deposition and tau phosphorylation; suppress neuroinflammation and oxidative stress	[63]
κ-carrageenan	Marine *red algae*	SH-SY5Y cells; LPS-BV2 microglia	—	In vitro incubation	—	Inhibit NF-κB-mediated neuroinflammation; activate Nrf2/HO-1	[67]
ι-carrageenan	Marine *red algae*	SH-SY5Y cells; LPS-BV2 microglia	—	In vitro incubation	—	Inhibit NF-κB-mediated neuroinflammation; activate Nrf2/HO-1	[68]
λ-carrageenan	Marine *red algae*	SH-SY5Y cells; LPS-BV2 microglia	—	In vitro incubation	—	Inhibit NF-κB-mediated neuroinflammation; activate Nrf2/HO-1; upregulate BDNF	[69]
Scallop polysaccharides	Marine scallop	AD model animals	—	—	—	Activate Nrf2/HO-1; reduce Aβ deposition and tau; improve learning and memory	[71]

**Footnote:** “—” denotes that complete experimental parameters (dose/concentration, administration route, or treatment duration) are absent from primary-source literature. Certain compounds are only qualitatively discussed in reviews, lacking full original in vitro/in vivo pharmacological data under AD-relevant pathological models.

**Table 2:** Summary of synthetic compounds against Alzheimer’s disease.

Compound	Source	Pharmacological Mechanism	Ref.
**Rivastigmine**	Fully synthetic compound	Reversible dual inhibitors of AChE and BChE	[11]
**Donepezil**	Fully synthetic small-molecule compound	Inhibition of AChE	[11]
**Galantamine**	Fully synthetic small-molecule compound	Reversible cholinesterase inhibitors	[11]
**Tacrine**	Fully synthetic small-molecule compound	Simultaneous inhibition of AChE) and BChE	[11]
**Memantine**	Fully synthetic small-molecule compound	Antagonist of the NMDAR	[12]
**Aducanumab**	Humanized recombinant monoclonal antibody	Anti-Aβ monoclonal antibody.	[14]
**Lecanemab**	Humanized recombinant monoclonal antibody	Anti-Aβ monoclonal antibody.	[13,14]
**Donanemab**	Humanized recombinant monoclonal antibody	Selectively binds to deposited amyloid-β (Aβ) plaques in the brain and promotes their clearance via immune-mediated mechanisms	[15]
**Verubecestat (MK-8931)**	Fully synthetic small-molecule BACE-1 inhibitor	Inhibits aberrant APP cleavage and reduces the production of Aβ peptides	[21]
**Tideglusib (NP-12)**	Fully synthetic small-molecule compound	Irreversible GSK-3β inhibitor	[28]
**GTS-21 (DMXBA)**	Fully synthetic small-molecule α7-nAChR agonist	α7-nAChR agonist.	[34]
**Nelonicline (ABT-126)**	Fully synthetic small-molecule α7-nAChR agonist	Selective α7 nicotinic receptor agonist	[49]

## 4. ADMET Analysis of Drugs for Alzheimer’s Disease

ADMET analysis serves as a foundational framework for assessing the druggability and safety profiles of candidate compounds. To systematically investigate the therapeutic potential of the 15 marine-derived compounds listed in Table 3, we conducted a comprehensive in silico evaluation using ADMETlab 3.0, drawing upon their detailed physicochemical and pharmacokinetic parameters [108,109]. This assessment thoroughly examined their drug-like properties and application prospects across critical dimensions, including physicochemical properties (molecular weight, lipophilicity, and aqueous solubility), absorption (Caco-2 permeability, P-glycoprotein interactions, and human intestinal absorption), distribution (plasma protein binding, blood–brain barrier penetration, and unbound fraction), metabolism (CYP450 inhibition and substrate specificity), excretion (clearance and half-life), and toxicity (hERG liability, Ames mutagenicity, and other safety endpoints). The results revealed marked diversity and heterogeneity among these compounds with respect to molecular size, hydrophobicity, solubility behavior, membrane permeability, protein binding affinity, metabolic stability, clearance kinetics, and inherent toxicity risks, highlighting both promising candidates and compounds with significant developability challenges.

### 4.1. Blood–Brain Barrier Permeability

For therapeutic agents targeting Alzheimer’s disease, effective penetration of the blood–brain barrier (BBB) is a critical prerequisite, as the primary pathological processes occur within the brain parenchyma. Candidate compounds must reach the central nervous system at sufficient concentrations to exert their pharmacological effects. In this data analysis, compounds predicted to possess high BBB penetration potential, specifically those denoted by the symbols “+++” and “++”, were prioritized as the most promising for further investigation in the context of Alzheimer’s disease therapy. According to the data in the table, the corresponding compounds with high BBB permeability are **compound 16** (+++) and **Homotaurine** (++). In contrast, **Verubecestat**, **Tideglusib**, **GTS-21**, **gracillin**, **Bryostatin 1**, **Manzamine A**, **Saxitoxin**, **Astaxanthin**, **Fucosterol**, **and phytoceramide** all exhibited “---”, while **Nelonicline** showed “+”, and **DHA and Phloroglucinol** displayed “--”, indicating extremely limited brain entry that would require structural modification or carrier-mediated delivery for improvement. Therefore, **compound 16 and Homotaurine** constitute the primary focus for subsequent pharmacodynamic evaluation and mechanistic studies, whereas the remaining compounds should be prioritized for BBB penetration enhancement before being considered for anti-AD drug development.

**Table 3:** ADMET analysis.

Parameters	Verubecestat	Tideglusib	GTS-21	Nelonicline	Gracillin	Compound 16	Bryostatin 1	Manzamine A	Saxitoxin	Astaxanthin	Phloroglucinol	Fucosterol	Phytoceramide	DHA	Homotaurine
MW	409.1	334.08	308.15	313.12	884.48	248.25	904.45	548.35	313.15	596.39	126.03	412.37	345.32	328.24	139.03
LogP	1.649	3.651	3.158	3.836	2.317	5.345	3.646	4.335	−0.915	4.375	0.329	7.696	4.275	6.843	−3.214
LogS	−3.233	−5.034	−4.021	−4.103	−4.713	−5.624	−5.317	−5.606	−0.884	−5.212	−1.413	−7.038	−3.857	−4.684	0.111
Caco-2	−4.828	−4.667	−4.698	−4.836	−6.388	−4.772	−5.484	−5.31	−5.708	−5.045	−5.065	−5.136	−5.184	−4.981	−6.118
Pgp-inh	--	+	++	---	---	++	+	---	---	-	---	---	---	--	---
Pgp-sub	+++	---	---	---	+	---	+++	---	+++	+++	---	---	--	---	++
HIA	---	---	---	---	---	---	---	---	-	---	---	---	++	---	+++
F (30%)	---	++	---	---	++	---	+++	---	++	+++	-	-	+++	---	--
BBB	--	---	--	+	---	+++	---	---	---	---	--	---	---	--	++
PPB	0.853	0.982	0.87	0.927	0.615	0.959	0.736	0.95	0.056	0.947	0.528	0.961	0.987	0.978	0.075
Fu	0.131	0.01	0.098	0.074	0.304	0.047	0.146	0.05	0.91	0.055	0.352	0.051	0.02	0.023	0.934
CYP1A2-inh	---	+++	+++	---	---	---	---	---	---	---	+++	---	---	--	---
CYP1A2-sub	---	+	+++	---	---	---	---	+++	---	+++	+++	---	---	---	---
CL	4.627	2.883	8.737	6.167	0.36	8.399	1.37	8.34	2.58	4.941	11.771	12.069	4.472	5.329	3.602
T_1/2_	0.643	0.816	0.632	1.159	3.709	0.177	1.992	0.719	2.091	0.885	1.332	0.545	1.175	0.27	1.629
hERG	0.341	0.248	0.427	0.371	0.026	0.074	0.009	0.992	0.218	0.046	0.146	0.129	0.285	0.621	0.021
Ames	0.755	0.691	0.279	0.651	0.916	0.351	0.998	0.227	0.84	0.89	0.477	0.086	0.064	0.968	0.176
ROA	0.774	0.098	0.562	0.641	0.009	0.286	0.475	0.997	0.84	0.833	0.464	0.094	0.042	0.016	0.094
FDAMDD	0.661	0.597	0.813	0.421	0.013	0.491	0.989	0.999	0.439	0.767	0.734	0.521	0.069	0	0.255
BCF	0.31	1.042	1.687	1.426	1.782	3.164	0.436	1.996	0.074	2.696	0.483	2.913	1.452	1.372	0.219

MW: molecular weight. LogS: the logarithm of the aqueous solubility value. LogP: the logarithm of the n-octanol/water distribution coefficient. Pgp-inh: the inhibitor of P-glycoprotein. Pgp-sub: the substrates of P-glycoprotein. HIA: human intestinal absorption. F (30%): the human oral bioavailability 30%. Caco-2: the permeability of human colon adenocarcinoma cell lines. BBB: the penetration of blood–brain barrier (BBB). PPB: plasma protein binding. Fu: the fraction unbound in plasma. CL: the clearance of a drug (mL/min/kg). T1/2: the half-life of a drug. hERG: the human ether-a-go-go-related gene. Ames: the Ames test for mutagenicity. ROA: the toxicity of rat oral acute. FDAMDD: the maximum recommended daily dose. BCF: the bioconcentration factor. For the classification endpoints, the prediction probability values are transformed into six symbols: 0–0.1 (---),0.1–0.3 (--), 0.3–0.5 (-), 0.5–0.7 (+), 0.7–0.9 (++), and 0.9–1.0 (+++). The data acquired from the ADMETLab 3.0 database. All parameters are in silico computational outputs from ADMETlab 3.0, not experimental measurements.

### 4.2. Physicochemical Properties

Based on the data in the table, an analysis of the 15 listed candidate compounds shows that their hERG values range from 0.009 to 0.992 and Ames values from 0.064 to 0.998, reflecting considerable variability in cardiac and mutagenic toxicity risks. To prioritize compounds with low-toxicity profiles, we set the criteria of hERG < 0.3 (low risk of QT prolongation) and Ames < 0.5 (low mutagenic potential). Under these criteria, the compounds that qualify include **compound 16**, **Phloroglucinol**, **Fucosterol**, ** phytoceramide**, **and Homotaurine**. Among these, **Homotaurine** stands out with high blood–brain barrier penetration (BBB ++), making it the most promising candidate for further anti-Alzheimer’s drug development. In contrast, **compound 16**, **Phloroglucinol**, ** Fucosterol**, **and phytoceramide** exhibit low or moderate BBB permeability (---, --, ---, and ---, respectively), and would require structural modification or carrier-assisted delivery to improve their brain entry before their pharmacological effects can be fully realized. Therefore, **Homotaurine** constitutes the primary focus for subsequent mechanistic studies and lead optimization, whereas the other four compounds should be prioritized for BBB penetration enhancement strategies. This screening provides a clear direction and a refined candidate pool for further investigation.

### 4.3. Absorption Properties

Based on a systematic evaluation of the metabolic stability and clearance characteristics of the 15 listed candidate compounds, we focused on three key parameters: CYP1A2-mediated inhibition and substrate potential (CYP1A2-inh and CYP1A2-sub), total clearance (CL), and elimination half-life (T1/2). For orally administered Alzheimer’s disease therapeutics, favorable metabolic profiles are essential to avoid drug–drug interactions and ensure predictable pharmacokinetics: low CYP1A2 inhibition and substrate liability (ideally denoted as “---”) minimize the risk of altered metabolism of co-administered drugs; moderate clearance (e.g., CL in the range of 2–8) prevents either too rapid elimination (leading to subtherapeutic exposure) or too slow elimination (causing accumulation and potential toxicity); and an appropriate half-life (e.g., T1/2 between 0.5 and 2.5 h) supports convenient dosing regimens while maintaining steady-state concentrations.

Analysis reveals substantial heterogeneity in the metabolic and elimination profiles of these compounds. With respect to CYP1A2 interactions, the majority of compounds (**Verubecestat**, **Nelonicline**, **gracillin**, **compound 16**, **Bryostatin 1**, **Saxitoxin**, **Fucosterol**, **phytoceramide**, **and Homotaurine**) exhibit “---” for both inhibition and substrate, indicating low risk. However, **Tideglusib and GTS-21** show strong inhibition (+++) and substrate activity (+++ or +), **Phloroglucinol** also shows +++ for both, **Manzamine A and Astaxanthin** are substrates (+++) despite no inhibition, and **DHA** shows moderate inhibition (--), highlighting potential metabolic liabilities that would require caution in polypharmacy settings. Regarding clearance, the values span a wide range from 0.36 (**gracillin**) to 12.069 (Fucosterol). Compounds with CL in the moderate range (2–8) include Verubecestat (4.627), **Tideglusib** (2.883), Nelonicline (6.167), **Saxitoxin** (2.58), Astaxanthin (4.941), phytoceramide (4.472), DHA (5.329), and Homotaurine (3.602), whereas **gracillin** (0.36) and **Bryostatin 1** (1.37) show low clearance, and compound 16 (8.399), Manzamine A (8.34), Phloroglucinol (11.771) and Fucosterol (12.069) exhibit high clearance. Half-life values are equally variable: T1/2 ranges from 0.177 (compound 16) to 3.709 (**gracillin**). Most compounds have half-lives between 0.5 and 2.5 h, with exceptions such as compound 16 (0.177, too short), **gracillin** (3.709, too long), and **Bryostatin 1** (1.992, acceptable but paired with low clearance).

Integrating the three criteria—low CYP risk (--- for both), moderate CL (2–8), and suitable T1/2 (0.5–2.5 h)—the compounds that met the requirements are **Verubecestat**, **Nelonicline**, **Saxitoxin**, **phytoceramide**, **and Homotaurine**. Among these, **Homotaurine** additionally exhibits high blood–brain barrier penetration (BBB ++), making it the most attractive candidate for further anti-AD development. **Verubecestat and Nelonicline** also show moderate BBB penetration (-- and +, respectively), but would benefit from further optimization to enhance brain entry. **Saxitoxin and phytoceramide**, despite excellent metabolic stability, have poor BBB permeability (---) and would require structural modification or carrier-mediated delivery to achieve therapeutic brain concentrations. In contrast, compounds with CYP liabilities or unfavorable CL/T1/2 values should be deprioritized or subjected to structural modification to mitigate metabolic risks. This screening provides a clear direction for prioritizing metabolically favorable candidates and highlights specific parameters that require optimization for the remaining compounds, ultimately guiding the selection of lead candidates with balanced pharmacokinetic and safety profiles.

### 4.4. Toxicity and Safety

Based on the comprehensive ADMET and safety profile analysis of the 15 candidate compounds, **Homotaurine** demonstrates the most balanced and outstanding characteristics across multiple key properties. It exhibits good potential for blood–brain barrier penetration (predicted value: ++), meets the basic drug-likeness criteria (MW: 139.03, LogP: –3.214), and shows outstanding predicted human intestinal absorption (HIA: +++), along with favorable oral bioavailability potential (F(30%): ++). In terms of metabolic stability, Homotaurine presents negligible CYP1A2 inhibition and substrate risks (both “---”), a moderate clearance rate (CL: 3.602) and a suitable half-life (T1/2: 1.629 h), indicating predictable pharmacokinetics with low drug–drug interaction potential. Regarding safety, it shows a very low hERG inhibition risk (0.021), low Ames mutagenicity (0.176), and a bioconcentration factor (BCF: 0.219) within a safe range, collectively indicating low cardiac toxicity, genotoxicity, and environmental accumulation risks. Moreover, its high unbound fraction (Fu: 0.934) suggests ample free drug available for pharmacological action, while its moderate plasma protein binding (PPB: 0.075) further supports favorable distribution.

In contrast, other compounds exhibit various shortcomings in critical parameters. **Compound 16**, despite possessing the highest BBB penetration (+++), suffers from extremely poor oral bioavailability potential (F(30%): “---”) and human intestinal absorption (HIA: “---”), along with a short half-life (0.177 h) and relatively high clearance (8.399), severely limiting its oral administration feasibility. **Phytoceramide** shows excellent F(30%) (+++) and good HIA (++), but faces very weak BBB penetration (“---”) and a relatively high BCF (1.452), while its low Fu (0.02) and high PPB (0.987) indicate limited free drug concentration. **Bryostatin 1,** although it achieves +++ in both F(30%) and HIA, has BBB penetration “---”, and despite a low hERG risk (0.009), exhibits extremely high Ames mutagenicity (0.998), making its genotoxicity risk non-negligible. **Verubecestat** shows moderate CL and T1/2, but its BBB penetration (“--”) and HIA (“---”) are suboptimal, and its high PPB (0.853) limits free fraction. **Saxitoxin** has excellent metabolic stability and a favorable half-life, but poor BBB (“---”) and very low Fu (0.056) restrict its brain availability. Among compounds meeting the dual drug-likeness criteria (MW ≤ 500 and LogP ≤ 5), **GTS-21**, **Saxitoxin**, **phytoceramide**, **and Homotaurine** all qualify, but only **Homotaurine** simultaneously possesses favorable BBB penetration, strong absorption properties, and an excellent safety profile; the others are limited by brain-targeting deficiencies or toxicity risks.

In summary, from the perspective of balancing brain targeting, oral absorption potential, metabolic stability, and comprehensive safety, **Homotaurine** emerges as the most promising candidate in this group due to its excellent BBB and HIA predictions, favorable drug-likeness parameters, moderate clearance, suitable half-life, low CYP liability, and low multiple toxicity risks. This analysis further confirms that for central nervous system drug development, balancing oral bioavailability with cardiac and genetic safety while achieving effective brain penetration remains a core challenge for most candidates. Subsequent research should prioritize targeted structural optimization of compounds such as **compound 16 and phytoceramide**, which excel in certain parameters but have shortcomings in safety or absorption, with the goal of ultimately identifying anti-AD candidates that combine favorable brain-targeting ability with oral administration feasibility.

## 5. Key Challenges on Marine Natural Products Against AD

Although marine natural products have shown great potential in the research against AD, and some compounds have entered preclinical or clinical studies, numerous critical challenges still exist, hindering their clinical translation and application.

### 5.1. Insufficient Blood–Brain Barrier Penetration

The BBB is a critical barrier for maintaining homeostasis in the central nervous system [110], preventing harmful substances from entering the brain parenchyma while simultaneously restricting the entry of most drugs into the brain for therapeutic action. Being macromolecules or highly polar compounds, most marine natural products (e.g., polysaccharides and polypeptides) cannot readily penetrate the BBB to reach the brain parenchyma. Their low intracerebral concentration fails to produce satisfactory anti-AD efficacy. Likewise, many small molecules such as terpenoids and alkaloids also possess weak BBB penetration [111], necessitating structural modification or formulation improvement for better brain delivery. For example, marine polysaccharides have high molecular weights and strong polarity, which hinders their penetration across the BBB. Their anti-AD effects mainly depend on the regulation of the gut–brain axis, with limited direct impacts on the central nervous system. Although carrier modification using liposomes, nanoparticles and other vehicles can enhance their BBB permeability, such approaches still suffer from low delivery efficiency and unvalidated safety. Liposomes modified with fucoidan have raised the drug delivery efficiency up to 78%, presenting a promising strategy to overcome the BBB penetration barrier. However, further optimization is still required.

### 5.2. Poor Pharmacokinetic Properties

Pharmacokinetic properties serve as critical indicators for evaluating druggability. Most marine natural products are characterized by low oral bioavailability, rapid metabolism and short half-life, which compromise their clinical efficacy. For instance, marine polyphenols and carotenoids are easily degraded by enzymes in the gastrointestinal tract, leading to extremely low oral bioavailability. Some terpenoids and alkaloids undergo rapid metabolism in vivo with short half-lives. Frequent administration is thus required to maintain effective plasma drug concentration, which increases the risk of adverse reactions. Furthermore, the absorption, distribution, metabolism and excretion (ADME) profiles of several marine natural products remain unclear, making it difficult to conduct rational dosage form design and optimize administration regimens [112]. As an example, the neuroprotective effects of sulfated polysaccharides present a non-linear pattern, and their optimal dose ranges need individualized assessment, which further complicates pharmacokinetic research. Structural modifications such as esterification and glycosylation can improve the pharmacokinetic performance of certain compounds, yet extensive studies are still needed to verify their efficacy and safety. Liposome encapsulation has increased the bioavailability of fucoidan from 12% to 65%, offering an effective approach to optimize the pharmacokinetic properties of marine natural products. Nevertheless, large-scale application of this technology still faces considerable challenges.

## 6. Conclusions and Future Perspectives

Marine natural products, including polysaccharides, terpenoids, macrolides, alkaloids, polyphenols-carotenoids, steroids-lipids and aminosulfonates, exert multi-target neuroprotective effects against Alzheimer’s disease (AD). They mitigate AD pathology via inhibiting Aβ aggregation and tau hyperphosphorylation, suppressing neuroinflammation and oxidative stress, restoring cholinergic function, promoting synaptic repair, and modulating the gut–brain axis.

Several marine-derived candidates have entered clinical studies: sodium oligomannate (GV-971) completed Phase III trials, bryostatin-1 reached Phase II, and homotaurine finished Phase II/III evaluations. However, most bioactive marine compounds remain confined to in vitro and pre-clinical animal experiments. Major translational barriers include poor blood–brain barrier permeability, unsatisfactory pharmacokinetic profiles, incomplete mechanistic evidence, low natural abundance, and efficacy gaps between animal models and human patients.

In silico ADMET profiling in this review indicates homotaurine as the most druggable candidate among tested molecules, with favorable brain penetration potential, oral absorption and low predicted toxicity. Most other marine agents show potent bioactivity but suffer from suboptimal brain exposure or metabolic properties, requiring structural modification or nanocarrier delivery.

Future work should focus on clarifying molecular targets of marine bioactive substances, optimizing BBB permeability and pharmacokinetic behaviors via structural modification or delivery systems, and strengthening translational validation. Interdisciplinary cooperation across marine science, medicinal chemistry, pharmacology and clinical medicine is required to build complete R&D pipelines and accelerate anti-AD drug development from marine resources.

## Data Availability

No new data were created or analyzed in this study.
